# Characterization of the FtsZ C-Terminal Variable (CTV) Region in Z-Ring Assembly and Interaction with the Z-Ring Stabilizer ZapD in *E*. *coli* Cytokinesis

**DOI:** 10.1371/journal.pone.0153337

**Published:** 2016-04-18

**Authors:** Kuo-Hsiang Huang, Aaron Mychack, Lukasz Tchorzewski, Anuradha Janakiraman

**Affiliations:** 1 Department of Biology, City College of CUNY, 160 Convent Avenue, MR 526, New York, NY, United States of America; 2 The Graduate Center of CUNY, 365 Fifth Avenue, New York, NY, United States of America; Centre National de la Recherche Scientifique, Aix-Marseille Université, FRANCE

## Abstract

Polymerization of a ring-like cytoskeletal structure, the Z-ring, at midcell is a highly conserved feature in virtually all bacteria. The Z-ring is composed of short protofilaments of the tubulin homolog FtsZ, randomly arranged and held together through lateral interactions. *In vitro*, lateral associations between FtsZ protofilaments are stabilized by crowding agents, high concentrations of divalent cations, or in some cases, low pH. *In vivo*, the last 4–10 amino acid residues at the C-terminus of FtsZ (the C-terminal variable region, CTV) have been implicated in mediating lateral associations between FtsZ protofilaments through charge shielding. Multiple Z-ring associated proteins (Zaps), also promote lateral interactions between FtsZ protofilaments to stabilize the FtsZ ring *in vivo*. Here we characterize the complementary role/s of the CTV of *E*. *coli* FtsZ and the FtsZ-ring stabilizing protein ZapD, in FtsZ assembly. We show that the net charge of the FtsZ CTV not only affects FtsZ protofilament bundling, confirming earlier observations, but likely also the length of the FtsZ protofilaments *in vitro*. The CTV residues also have important consequences for Z-ring assembly and interaction with ZapD in the cell. ZapD requires the FtsZ CTV region for interaction with FtsZ *in vitro* and for localization to midcell *in vivo*. Our data suggest a mechanism in which the CTV residues, particularly K380, facilitate a conformation for the conserved carboxy-terminal residues in FtsZ, that lie immediately N-terminal to the CTV, to enable optimal contact with ZapD. Further, phylogenetic analyses suggest a correlation between the nature of FtsZ CTV residues and the presence of ZapD in the β- γ-proteobacterial species.

## Introduction

The assembly of the tubulin-like GTPase, FtsZ, at midcell is a conserved, essential feature of bacterial cytokinesis in most species. Polymers of FtsZ form a cytokinetic ring-like structure called the Z-ring that provides a scaffold for the recruitment of other division proteins and plausibly contributes towards the generation of a constriction force on the inner cell membrane [1–5].

In its GTP-bound state, FtsZ assembles into single stranded polymers (protofilaments) [6–10]. *In vitro*, FtsZ protofilaments can assemble with various geometries including bundles, sheets or rings but the *in vivo* relevance of these structures is unclear [11,12]. Super-resolution microscopy of *E*. *coli* cells have revealed the Z-ring to consist of short, randomly arranged, overlapping protofilaments that are held together by lateral interactions [13–16].

**Fig 1:**
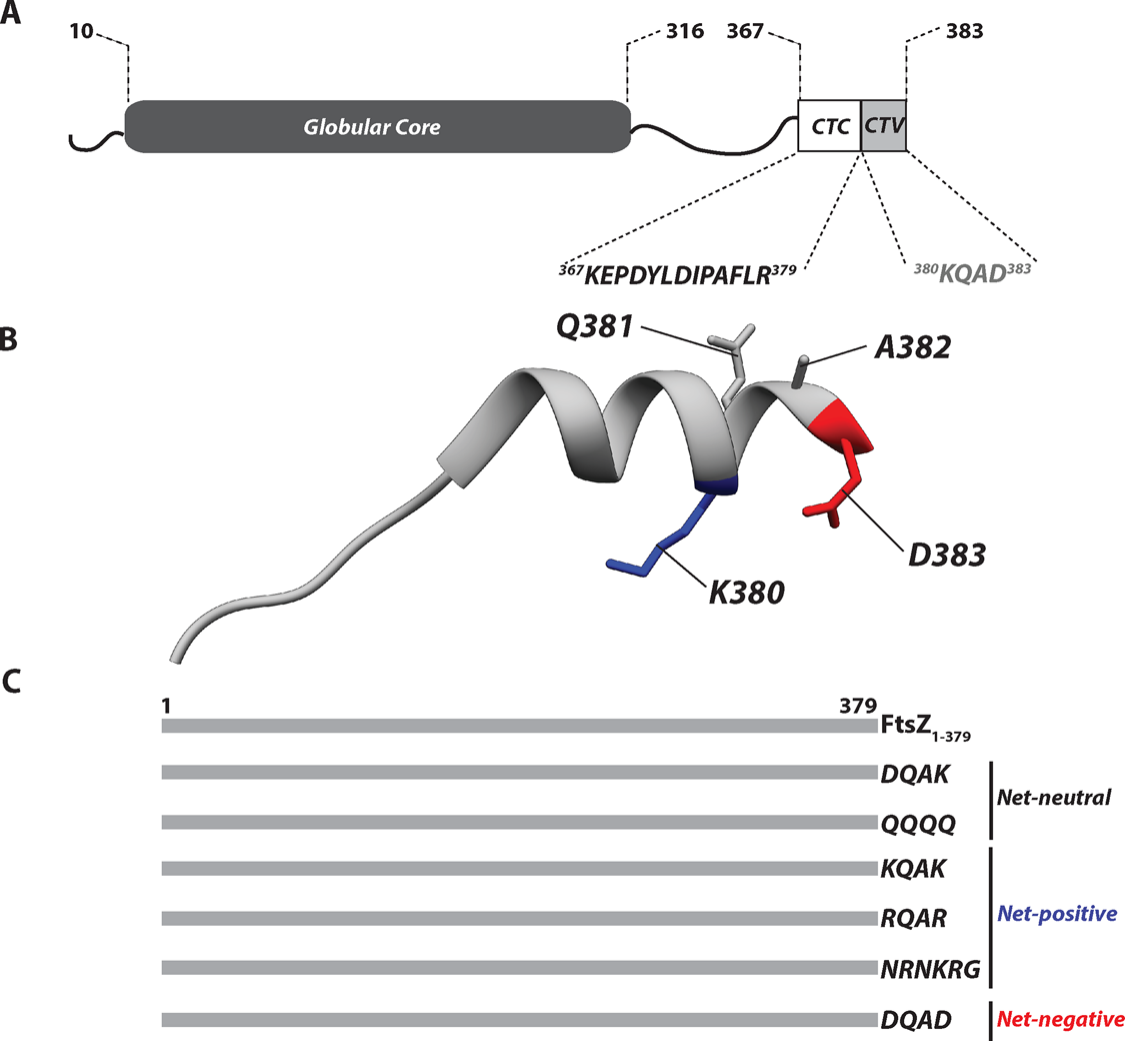
FtsZ domain structure, FtsZ C-terminal tail (CTT) structure and FtsZ C-terminal variable (CTV) mutant constructs. **A.** Domain organization of *E*. *coli* FtsZ: an unstructured 10 residues at the N-terminal end (squiggly line), a conserved globular core domain containing the nucleotide binding and hydrolysis residues, a flexible variable linker about 50 residues long (squiggly line), and a conserved carboxy terminal peptide (CCTP) which contains both a constant region of ~13 residues (CTC) and a variable region of 4 residues (CTV). **B.** Structural model of the FtsZ C-terminal residues 367–383 (PDB 1F47) [29]. In a X-ray crystal structure complex with the essential division protein ZipA, the 17 residue FtsZ CCTP binds as an extended β-strand followed by an α-helix. The CTV residue side-chains are identified in the α-helix: K380 (blue), Q381 and A382 (gray) and D383 (red). **C.** Schematic of the FtsZ CTV mutant constructs used in the study, not drawn to scale.

The FtsZ monomer comprises four domains: an unstructured poorly conserved region of ~10 residues at the extreme N-terminus; a highly conserved globular core containing the GTP binding and hydrolytic functions; a disordered flexible linker that is poorly conserved in length and sequence among species; and the C-terminal conserved peptide (CCTP) which contains two sub-regions: a conserved C-terminal constant region (CTC) and a C-terminal variable region (CTV) (Fig 1) [17–19]. *In vitro*, the globular core alone is sufficient for formation of FtsZ protofilaments [20,21]. But, recent work has implicated the flexible linker and FtsZ CTV sequences to be key determinants of the end-to-end and lateral interactions of FtsZ *in vitr*o implying a role for these domains in the architecture of FtsZ assemblies in the cell [17,18,22]. Most known stabilizers and destabilizers of FtsZ interact with the CCTP, which serves as a dock for proteins that regulate FtsZ-ring assembly dynamics. Such proteins include the essential FtsZ membrane tethers FtsA and ZipA, positional regulators MinC and SlmA, the conserved protease ClpX, and the Z-ring stabilizer ZapD [23–28]. The structure of CCTP bound to ZipA in *E*. *coli* and FtsA in *T*. *maritima* have been solved [29,30]. Although the CCTP in each case contains a helical segment starting at a conserved proline, the extended structures are not identical, suggesting that the CCTP is capable of acquiring a variety of structures possibly to enable interactions with varied binding partners [30,31].

In *E*. *coli* and related species, Z-ring assembly is thought to initiate through the formation of an unstable proto-ring that consists of FtsZ, FtsA, and ZipA [32]. In addition to attaching FtsZ filaments to the membrane, both FtsA and ZipA contribute to the integrity of the Z-ring, ZipA by increasing lateral associations among FtsZ protofilaments [21,33,34]. The Z-ring is subsequently stabilized through interactions with several FtsZ-ring associated proteins (Zaps): ZapA, ZapB, ZapC, and ZapD. The Zaps localize to midcell early during cytokinesis and exhibit functional overlap in affecting the assembly dynamics of FtsZ [25,28,35–41]. Although deletion of a single *zap* gene shows only modest defects in division and Z-ring morphology in WT cells, the phenotypes are exacerbated in cells lacking two or more Zap proteins indicating their important contributions to the architecture and function of the Z-ring. Notably, though the Zaps are functionally redundant they are not homologous proteins, and only ZapA is widely conserved [35].

ZapA interacts directly with FtsZ and promotes both longitudinal and lateral interactions of FtsZ polymers *in vitro* with a concomitant reduction in FtsZ GTPase activity [37,38,42,43]. Both ZapA and ZapB, which is recruited to the Z-ring by ZapA, are implicated in condensing FtsZ polymers into a tight-pitched ring at midcell, and influencing cell constriction [44–47]. The structure of ZapC has recently been solved by two groups independently, and evidence suggests that it employs an extensive binding surface to interact with FtsZ [48–50]. Less is known about the specific contributions of ZapD to Z-ring architecture and function.

Towards our long-term goal of characterizing the modulatory roles of the Zap proteins in Z-ring dynamics, we sought to understand their functional overlap in stabilizing Z-ring assembly in *E*. *coli* and related species. The net-charge of amino acid residues in the FtsZ CTV has been shown to affect FtsZ lateral interactions *in vitro* independent of modulatory proteins [22]. Since the net-charge of the FtsZ CTV varies from species to species, we sought to test the hypothesis, first suggested by Buske and Levin, that modulatory proteins may act by compensating for CTV charge variations via their interactions with FtsZ CCTP. We focused on the Z-ring stabilizer ZapD as prior studies pointed to FtsZ CCTP to be its binding site [25]. *E*. *coli* FtsZ mutants with varying net-charges on their CTV were generated and assayed for their interactions with ZapD using a variety of methods including yeast-two hybrid, co-sedimentation assays, bundling, complementation, and in their ability to recruit ZapD to midcell *in vivo*. Our results indicate that FtsZ CTV residues, in particular K380, are important for interaction with ZapD. In addition, FtsZ CTV residues not only play a critical role in defining the lateral interaction potential of FtsZ assembly, confirming an earlier report, but also likely impact the end-to-end associations of FtsZ monomers *in vitro*.

## Materials and Methods

### Strains, Plasmids, and Growth Conditions

All strains and plasmids used in this study are listed in Tables 1 and 2. Plasmids were introduced into bacterial and yeast strains by electroporation and chemical transformation, respectively. *E*. *coli* strains were grown in LB (0.5% NaCl) broth or agar plates with appropriate antibiotics at 30°C unless otherwise mentioned. Antibiotics were applied at the following concentration: ampicillin, 100 μg/ml; kanamycin, 50 μg/ml; spectinomycin, 100 μg/ml (in LB) and 40 μg/ml in LB no salt (LBNS); and tetracycline, 12.5 μg/ml. Yeast strains were maintained in YPD media at 30°C, and upon transformation with pDEST-AD—or pDEST-BD fusion vectors (Arabidopsis Biological Resources Center) were grown in YNB complete medium (MP Biomedicals) lacking Leucine (-Leu) or Tryptophan (-Trp), respectively. Appropriate pairs of transformants were mated on YPD media at room temperature, and diploid yeast strains were grown in selective YNB broth (-Leu -Trp) at 30°C.

**Table 1:** Bacterial and yeast strains used in the study.

Strains and plasmids	Description	Source or Reference^a^
*E*. *coli*		
MG1655	K12 F^-^ λ^-^ *ilvG*^-^ *rfb*-50 *rph*-1	Laboratory collection
MGZ84	MG1655 *leu*::*Tn10 ftsZ84* (Ts)	Manjula Reddy
C41(DE3)	*F*^-^ *ompT hsdSB (rB*^-^ *mB*^-^*) gal dcm (DE3)*	Petra Levin
TB28	MG1655 *ΔlacIZYA*::*frt*	[62]
AMZ84	TB28 *leu*::*Tn10 ftsZ84* (Ts)	
KHH72	C41(DE3) pET28b-*his10x-smt3-zapD*	
KHH216	MG1655 pNG162	
KHH242	MG1655 pNG162-*ftsZ*	
KHH243	MG1655 pNG162-*ftsZ*_*DQAD*_	
KHH317	MG1655 pNG162-*ftsZ*_*1-379*_	
KHH351	MGZ84 pNG162-*ftsZ*	
KHH352	MGZ84 pNG162-*ftsZ*_*1-379*_	
KHH353	MGZ84 pNG162	
KHH356	MGZ84 pNG162-*ftsZ*_*DQAD*_	
KHH357	MGZ84 pNG162-*ftsZ*_*DQAK*_	
KHH358	MGZ84 pNG162- *ftsZ*_*QQQQ*_	
KHH367	MGZ84 pNG162-*ftsZ*_*KQAK*_	
KHH368	MGZ84 pNG162-*ftsZ*_*RQAR*_	
KHH369	MGZ84 pNG162-*ftsZ*_*NRNKRG*_	
KHH373	AMZ84 pNG162-*ftsZ* pDSW208-*zapD-gfp*	
KHH379	AMZ84 pNG162-*ftsZ*_*1-379*_ pDSW208-*zapD-gfp*	
KHH384	AMZ84 pNG162 pDSW208-*zapD-gfp*	
KHH389	AMZ84 pNG162-*ftsZ*_*DQAK*_ pDSW208-*zapD-gfp*	
KHH391	AMZ84 pNG162-*ftsZ*_*QQQQ*_ pDSW208-*zapD-gfp*	
KHH392	AMZ84 pNG162-*ftsZ*_*KQAK*_ pDSW208-*zapD-gfp*	
KHH395	AMZ84pNG162-*ftsZ*_*NRNKRG*_ pDSW208-*zapD-gfp*	
KHH397	TB28 pNG162-*ftsZ* pDSW208-*zapD-gfp*	
KHH401	AMZ84 pNG162-*ftsZ*_*RQAR*_ pDSW208-*zapD-gfp*	
*S*. *cerevisiae*		
PJ69-4A	*MATa trp1-901 leu2-3*,*112 ura3-52 his3-200 gal4∆ gal80∆ LYS2*::*GAL1-HIS3 GAL2-ADE2 met2*::*GAL7-lacZ*	Beate Schwer
SL3004	*MATα trp1-901 leu2 ura3 his3 gal4 gal80 lys2-801 ade2-101*	Sandra Lemmon
KHH-Y27	SL3004 pDEST-GADT7-*ftsZ*	
KHH-Y28	SL3004 pDEST-GADT7-*ftsZ*_*1-379*_	
KHH-Y29	SL3004 pDEST-GADT7-*ftsZ*_*DQAD*_	
KHH-Y30	SL3004 pDEST-GADT7-*ftsZ*_*KQAK*_	
KHH-Y31	SL3004 pDEST-GADT7-*ftsZ*_*QQQQ*_	
KHH-Y33	SL3004 pDEST-GADT7	
KHH-Y34	PJ69-4A pDEST-GBKT7-*zapD*	
KHH-Y35	PJ69-4A pDEST-GBKT7	
KHH-Y36	SL3004 pDEST-GADT7-*ftsZ*_*DQAK*_	
KHH-Y56	SL3004 pDEST-GADT7-*ftsZ*_*RQAR*_	
KHH-Y57	SL3004 pDEST-GADT7-*ftsZ*_*NRNKRG*_	

^a^ This study unless otherwise mentioned.

**Table 2:** Plasmids used in the study.

Plasmid	Description	Source or Reference^a^
pAM1	pNG162-*ftsZ*_*RQAR*_	
pAM2	pNG162-*ftsZ*_*NRNKRG*_	
pAM3	pET-21b(+)-*ftsZ*_*RQAR*_	
pDEST-GADT7	pGADT7 derived vector, *attR1 Cm*^*R*^ *ccdB attR2*	ABRC^b^
pDest-GADT7-*ftsZ*		[25]
pDEST-GBKT7	pGBKT7 derived vector, *attR1 Cm*^*R*^ *ccdB attR2*	
pDSW208	pDSW204-MCS-*gfp*, Amp^R^	[63]
pET21b(+)	pBR322 ori, Amp^R^	P.A. Levin
pET-21b(+)-*ftsZ*_1-379_	pET-21b(+)-*ftsZ*_1-379_	[22]
pET28b-His10-Smt3	pBR322 ori pT7-*his10*-*smt3*, Kan^R^	S. Shuman
pKHH1	pDEST-GADT7-*ftsZ*_*1-379*_	
pKHH2	pDEST-GADT7-*ftsZ*_*DQAD*_	
pKHH3	pDEST-GADT7-*ftsZ*_*KQAK*_	
pKHH4	pDEST-GADT8-*ftsZ*_*DQAK*_	
pKHH5	pDEST-GADT7-*ftsZ*_*QQQQ*_	
pKHH7	pET-21b(+)-*ftsZ*_*DQAD*_	
pKHH8	pET-21b(+)-*ftsZ*_*KQAK*_	
pKHH9	pET-21b(+)-*ftsZ*_*DQAK*_	
pKHH11	pNG162-*ftsZ*	
pKHH12	pNG162-*ftsZ*_1-379_	
pKHH13	pNG162-*ftsZ*_*DQAD*_	
pKHH14	pNG162-*ftsZ*_*KQAK*_	
pKHH15	pNG162-*ftsZ*_*DQAK*_	
pKHH16	pNG162-*ftsZ*_*QQQQ*_	
pKHH17	pDEST-GADT7-*ftsZ*_*RQAR*_	
pKHH18	pDEST-GADT7-*ftsZ*_*NRNKRG*_	
pKHH19	pDSW208-*zapD-gfp*	
pLT1	pET-21b(+)-*ftsZ*_*QQQQ*_	
pPJ2	pET-21b(+)-*ftsZ*	[22]
pPJ6	pET-21b(+)-*ftsZ*_*NRNKRG*_	”
pNG162	pSC101, Spec^R^	[64]

^a^ This study unless otherwise mentioned.

^b^ Arabidopsis Biological Resources Center.

### Plasmid Construction

All primers used in the study are listed in S1 Table. Plasmid pNG162 expressing FtsZ WT or CTV mutant proteins under the control of an IPTG-inducible promoter were constructed by amplifying *ftsZ* using the NcoI *ftsZ* forward primer and HindIII (or SalI) *ftsZ* reverse primer from the pET21b (+)—*EcftsZ* or pET21b (+)—*EcftsZCTVB* plasmids. The PCR product was digested by NcoI/HindIII or NcoI/BamHI and ligated into pNG162 vector treated with the same restriction enzymes. After PCR amplification with *ftsZ* forward GW and *ftsZ* (or one of *ftsZ*_CTV_ mutants) reverse GW primer, plasmids pDEST-GADT7-FtsZ or FtsZ_CTV_ mutants were cloned by Gateway recombination BP and LR reactions (Life Technologies). Plasmid pDSW208-*zapD-gfp* was constructed by amplifying *zapD* using the SacI *zapD* forward primer and SalI-*yacF* reverse primer from pTrc99a-*zapD*. The PCR product was digested by SacI/SalI and ligated into the pDSW208 vector treated with the same restriction enzymes. Resulting clones were verified by Sanger sequencing (Genewiz).

### Construction of Strains for Protein Expression and Purification

Using EcFtsZ1Fwd-pET21b primer with varying FtsZ reverse primers, all *ftsZ* CTV variants were amplified by PCR and the products were digested by NdeI in combination with BamHI or HindIII or SalI restriction enzymes and cloned into the same sites of pET21b (+) vector. After sequence verification, clones were transformed into C41/DE3 to express FtsZ CTV mutant proteins.

### Yeast-Two-Hybrid (Y2H) Assay

Diploid yeast were grown in YNB (-Leu-Trp) broth at 30°C for ~20 hours until OD_660_ = 0.5–1.0 and β-galactosidase measurements were made using the Y2H assay kit (ThermoFisher) essentially as described by the manufacturer. Briefly, 175 μls of cell suspension was diluted into an equal volume of YNB (-Leu-Trp) broth and mixed with 175 μls of 2X β-galactosidase assay buffer and 175 μls of Y-PER yeast protein extraction reagent. After incubating the mixtures at 37°C for ~25 minutes, reactions were stopped by adding 300 μls 1 M Na2CO3. Cell debris was removed by centrifugation and the absorbance of the supernatants was measured at 420 nm. Miller units were calculated based on the formula (1000 x A_420_)/(T x V x OD_660_) where T and V relate to reaction times and volumes.

### Protein Expression and Purification

Plasmid pET28b-His10-Smt3-ZapD was expressed in C41/DE3 cells and ZapD was purified as described previously [25]. FtsZ and FtsZ CTV mutants in C41/DE3 cells were grown to OD_600_ = 0.4 for 2–3 hours at which time IPTG was added to a final concentration of 0.7 mM and allowed to continue growth for an additional 2–3 hours. Proteins were extracted and purified by adding saturated ammonium sulfate as described [51]. All FtsZ CTV variants were purified at a 25% saturated ammonium sulfate cutoff except for the DQAD mutant, for which 20% ammonium sulfate cutoff was used.

### Sedimentation Assay

Purified FtsZ or FtsZ CTV mutants (5 μM) were combined with purified ZapD (1 or 5 μM) in our standard FtsZ polymerization buffer (50 mM K-MOPS; pH 6.5, 50 mM KCl, 2.5 mM MgCl2) containing 3 μM BSA, unless otherwise mentioned. Polymerization was initiated by addition of GTP (1 mM) to the assembled reaction above. Reaction mixtures (100 μls) were processed at room temperature and products were recovered by centrifugation using a TLA100.2 rotor at 80,000 rpm for 10 mins. An 81-μl aliquot of the supernatant was collected to which 27 μls of 4X loading dye was added for later analysis. The rest of the supernatant was carefully discarded, and the pellets were resuspended in 100 μls of the polymerization buffer and incubated at 65°C for 10 mins followed by addition of 33 μls of 4X loading dye. The supernatants and pellets were resolved in a 12.5% SDS-PAGE gel (Bio-Rad) and band intensities measured using Image J (NIH).

### Quantitative Immunoblotting

Growth conditions are described in the respective figure legends. Whole cell protein preparations were separated on 12.5% SDS-PAGE gels and transferred to nitrocellulose membranes. Blots were processed and analyzed essentially as described [25]. Briefly, FtsZ, FtsZ84, and FtsZ CTV mutant protein levels from *E*. *coli* strains were detected with an anti-FtsZ rabbit polyclonal antibody (Genscript) at 1:10,000 dilution, and ZapD-GFP was detected with an anti-GFP rabbit polyclonal antibody (Life Technologies) at 1:1000 dilution. RpoD (Sigma70) was detected with an anti-RpoD mouse monoclonal antibody (BioLegend) at 1:1000 dilution. Infrared fluorescence IRDye® secondary antibodies 800CW (Goat anti-rabbit) or 680RD (Goat anti-mouse) (LI-COR) were used at 1:20,000 dilutions. Bands were visualized using a LI-COR Odyssey CLx imager and intensities were measured using ImageStudio software (LI-COR).

### Transmission Electron Microscopy

FtsZ polymerization reactions were performed essentially as described above but without BSA. After 5 mins at room temperature a 10-μl aliquot was placed on a carbon-coated copper grid, negatively stained with 2% uranyl acetate for 5–10 secs, and wicked dry. Images were collected on a JEOL 2100 Lab6 TEM operated at 200kV with 30 pA/cm^2^ current density and recorded on a US1000 XP1 camera at a nominal magnification of X 30,000, unless otherwise mentioned.

### Spot Viability Assays

Overnight cultures of *ftsZ84* (Ts) with FtsZ or different FtsZ CTV mutants expressed *in trans* were grown in LB with 0.2% glucose and appropriate antibiotics at 30°C. Overnight cultures were spun, washed, and normalized to OD_600_ = 1.0 in LB or LBNS. Cell suspensions were serially diluted from 10–1 to 10–6 and 3 μls from each dilution was spotted on LB or LBNS plates with appropriate antibiotics plus 1 mM IPTG, and grown at 30°C or 42°C for ~24 hrs, at which point the plates were imaged (Syngene Gel-Doc system).

### Fluorescence Microscopy

Overnight cultures of *ftsZ84* (Ts) cells expressing FtsZ or FtsZ CTV mutants and/or ZapD *in trans* were grown as described in the figure legends and imaged by phase or fluorescent microscopy on 1.5% agarose pads using a Nikon TiE microscope. Slide temperature was maintained at 42°C using a TC-500 temperature controller (20/20 Inc). ObjectJ (NIH) was used to measure cell lengths and ZapD-GFP midcell localization from images as described [25].

For immunofluorescence studies, overnight cultures grown in LB were subcultured to OD_600_ = ~0.08 in LB in the presence of spectinomycin at 30°C and grown till OD_600_ = ~0.2, at which point cells were shifted to 42°C in LBNS media with 1 mM IPTG for 3 doublings (~1 hr 15 mins). Cells were fixed for immunofluorescence studies essentially as described [52]. FtsZ-rings were imaged using an anti-FtsZ rabbit polyclonal primary antibody (Genscript) at 1:10,000 dilution and a Texas-Red conjugated anti-rabbit secondary antibody at 1:50 dilution.

### Bioinformatics

FtsZ and/or ZapD homolog sequences from 427 species of α-, β- and γ- proteobacteria with fully sequenced genomes were collected from the NCBI Microbial Genomes Resources database (http://www.ncbi.nlm.nih.gov/genomes/MICROBES/microbial_taxtree.html). Sequences were analyzed by BLAST using the *E*. *coli* MG1655 FtsZ (NP_414637) and ZapD (NP_414644) sequences as queries. To identify potential *zapD* homologs sequences that meet the following criteria were chosen: ≥ 80% ORF coverage, ≥ 40% similarity and ≥ 20% identity compared to *E*. *coli* MG1655 ZapD sequence. The FtsZ C-terminal structure was extracted from PDB (1F47), and analyzed with PyMOL software (Schrödinger, LLC) [29].

## Results and Discussion

### FtsZ CTV Residues Contribute to the Interaction with ZapD in Yeast

Previous data from our lab indicated that ZapD interacts with the FtsZ CCTP in a PIP (protein-protein interaction) assay in yeast [25]. Briefly the PIP assay probes for interactions between two proteins when one protein is fused to a fluorophore and another to the reoviral scaffolding protein, μNS, which forms large focal inclusions in yeast [53]. Interactions between the query protein and a likely binding partner can be identified by visual screens for fluorescent foci in yeast. Notably, a GFP-ZapD fusion failed to form fluorescent foci with FtsZ lacking CCTP, but showed interactions with FtsZ residues 374–383 alone suggesting that FtsZ CCTP was necessary and sufficient for interaction with ZapD in yeast [25]. Furthermore, ZapD failed to bind and bundle FtsZ lacking the last 11 amino acids in a co-sedimentation assay *in vitro* suggesting the requirement of the CCTP sequences in binding ZapD [48].

FtsZ CTV regions are variable in length and sequence among species and consequently vary in their net-charge [22]. As the net charge of this region has been shown to have a dramatic impact on FtsZ lateral association potential [22], and ZapD mediates lateral bundling of FtsZ protofilaments, we sought to examine how the net charge content of the *E*. *coli* CTV residues contributes to the interaction with ZapD. To this end we first used a Y2H assay to examine the interactions between ZapD and either WT FtsZ, an FtsZ mutant in which the CTV has been removed (FtsZ_1-379_), and several FtsZ mutant derivatives (Fig 1) in which the native *E*. *coli* CTV sequence (KQAD) were replaced by (i) a net-neutral (DQAK or QQQQ) or (ii) a net-positive (RQAR or KQAK) or (iii) the *B*. *subtilis* CTV sequence (NRNKRG), which carries a net-positive CTV but differs in length, in the number of basic residues, and in their spacing compared to the KQAK and RQAR constructs or (iv) a net-negative (DQAD) CTV. We find that ZapD interacts with WT FtsZ and FtsZ CTV net-neutral (DQAK or QQQQ) or net-positive (RQAR or NRNKRG) variants (Table 3). In contrast, ZapD failed to interact with FtsZ lacking the CTV residues (FtsZ_1-379_), or with FtsZ CTV net-positive variant KQAK or net-negative variant DQAD (Table 3). All protein fusions were expressed stably in yeast except the KQAK variant, suggesting that the lack of interaction of ZapD with FtsZ CTV bearing KQAK residues was likely due to the instability of the fusion protein in yeast (S1 Fig). The protein-protein interaction studies suggest that the FtsZ CCTP plays a critical role in the interaction with ZapD in *E*. *coli*, and that the net-charge content of the FtsZ CTV residues maybe important for this association.

**Table 3:** A GAL4 BD-ZapD^a^ fusion fails to interact with FtsZ lacking the CTV residues in a yeast two-hybrid assay.

GAL4 AD fused protein^b^	β-galactosidase activity^c^ ± SD^d^
_^e^	40.9 ± 22.0
FtsZ	355.2 ± 56.9
FtsZ_1-379_	48.7 ± 16.7
FtsZ_DQAK_	170.5 ± 81.7
FtsZ_QQQQ_	114.9 ± 31.8
FtsZ_KQAK_	39.5 ± 17.3
FtsZ_RQAR_	155.1 ± 16.5
FtsZ_NRNKRG_	105.6 ± 10.4
FtsZ_DQAD_	50.5 ± 28.5

^a, b^ BD = DNA binding domain and AD = Activation domain.

^c^ β-galactosidase activity is in Miller units and represents mean activity of 10 independent trials.

^d^ SD = Standard Deviation.

^e^ AD vector alone.

### ZapD Fails to Interact with FtsZ Lacking the CTV Residues *In Vitro*

To test direct evidence of the interactions between FtsZ and the FtsZ CTV mutants with ZapD *in vitro*, we used purified proteins and conducted co-sedimentation assays. As mentioned earlier, our results indicate that ZapD fails to bind and bundle FtsZ lacking the CCTP residues suggesting their importance in mediating the ZapD/FtsZ interaction [48]. Our data indicate that ZapD promotes polymer assemblies of net-neutral, and specific net-positive FtsZ CTV mutant proteins (KQAK and RQAR) but fails to enhance polymerization of FtsZ lacking the CTV sequences, FtsZ with a net-negative CTV (DQAD), or FtsZ containing a *B*. *subtilis* CTV sequence (NRNKRG).

**Fig 2:**
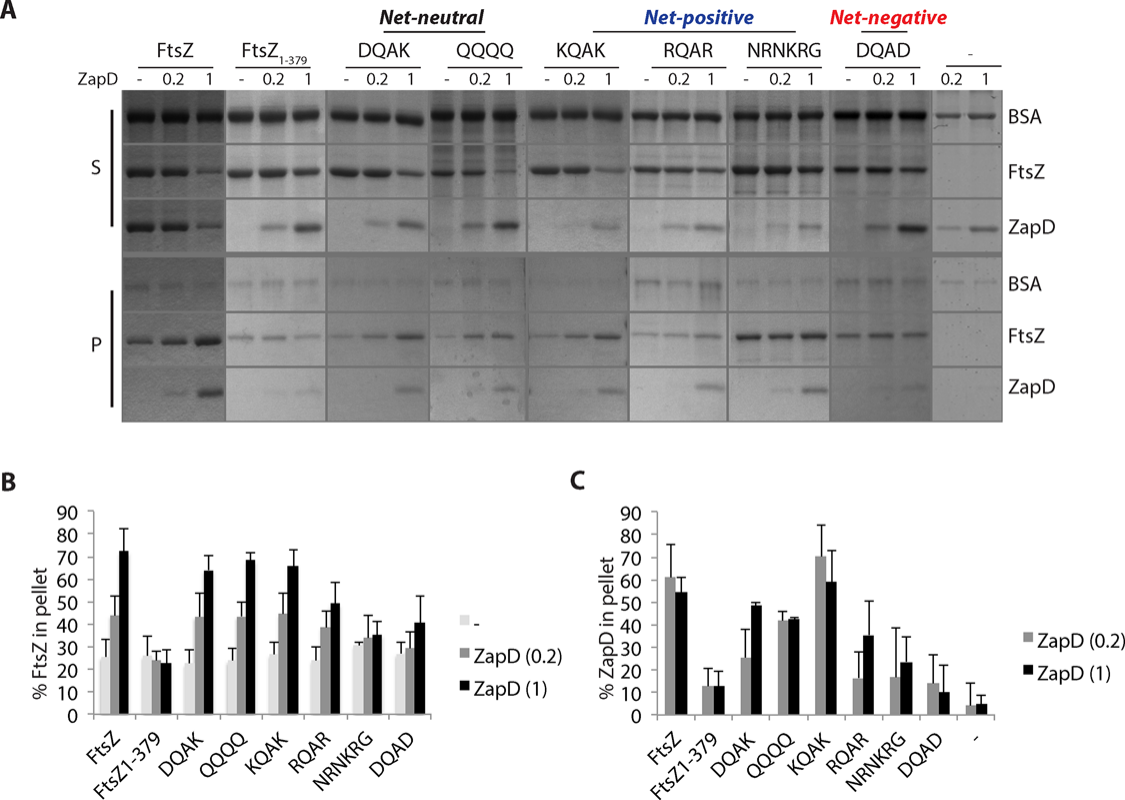
Sedimentation reactions of purified FtsZ and FtsZ CTV mutant proteins with ZapD. **A.** FtsZ and FtsZ CTV mutants (5 ∝M) were incubated alone or combined with purified ZapD at 1:0.2 or 1:1 ratios in a polymerization buffer (50 mM K-MOPS; pH 6.5, 50 mM KCl, 2.5 mM MgCl2, and 1 mM GTP) containing 3 ∝M BSA. Reactions were processed as outlined in the Materials and Methods section in the main text. Equivalent aliquots (5 ∝l) of pellet (bottom panel) and supernatants (top panel) were resolved on a 12.5% SDS-PAGE gel and stained with SimplyBlue SafeStain (Invitrogen). A representative gel image of three independent experiments is shown. **B.** The amounts of FtsZ or FtsZ CTV mutant proteins present in the pellet fractions in reactions with or without ZapD are reported as a percentage. The average numbers and standard deviation bars are from at least three independent experiments. Of note, FtsZ CTV containing NRNKRG sequences show the highest pelletable amounts of FtsZ under the experimental conditions of this study. **C.** The amounts of ZapD protein present in the pellet fractions in reactions with FtsZ or FtsZ CTV mutants are reported as a percentage. The average numbers and standard deviation bars are from at least three independent experiments.

When incubated with GTP, 26 ± 8% of WT FtsZ and similar amounts of FtsZ_1-379_ and other FtsZ CTV variants, except the NRNKRG variant were present in the pellet (Fig 2). It has been previously reported that an *E*. *coli* FtsZ chimeric construct containing a *B*. *subtilis* CTV sequence (NRNKRG) shows enhanced bundling compared to *E*. *coli* FtsZ [22]. In the presence of ZapD, ~2–3 fold increases in amounts of WT FtsZ were noted together with ~55–62% of ZapD recovered in the pellet. ZapD alone was barely detectable after sedimentation in the presence of GTP (Fig 2). These data are consistent with an increase in the sedimentable WT FtsZ polymer mass. However, no significant changes in pelletable amounts of FtsZ without the CTV region (FtsZ_1-379_) were observed with or without ZapD, nor did significant amounts of ZapD co-sediment with FtsZ_1-379_ (Fig 2).

In the presence of ZapD, net-neutral (DQAK, QQQQ) or net-positive (KQAK, RQAR) FtsZ CTV mutants showed ~2–3 fold increases in pelletable amounts of mutant FtsZ proteins similar to WT FtsZ, consistent with increases in sedimentable FtsZ polymeric assemblies. Additionally, ZapD co-pelleted with the FtsZ variants in more or less similar amounts in reactions where ZapD and FtsZ were present in equimolar concentrations (Fig 2). As expected, in the presence of GTP, increased amounts of the NRNKRG mutant was present in the pellet compared to WT FtsZ, even at lower concentrations of the variant protein (Fig 2, S2 Fig). Addition of ZapD did not increase the fraction of sedimentable NRNKRG polymers though ZapD is recovered in the pellet in these reactions (Fig 2, S2 Fig). This suggests that while ZapD can bind the NRNKRG CTV, it does not result in a functional interaction. Another possibility is that the ZapD/NRNKRG interaction is non-specific, and the presence of ZapD in the pellet results from crowding effects of NRNKRG polymeric bundles. Lastly, an FtsZ variant that carries a net-negative CTV (DQAD) failed to show any significant increases in the sedimentable amounts of the DQAD mutant in the presence of ZapD nor did ZapD co-pellet in any appreciable amount in these reactions even at equimolar ratios (Fig 2). These results indicate that FtsZ CTV residues make a critical contribution in ZapD-mediated FtsZ polymerization and are discussed in concert with the TEM results below.

### ZapD Does Not Promote Bundling of FtsZ Mutants Lacking CTV Sequences *In Vitro*

In order to ascertain whether an increase in sedimentable amounts of FtsZ or FtsZ CTV mutants either alone, or in the presence of ZapD, corresponds to morphological changes in FtsZ polymeric assemblies, we visualized the products of the polymerization reactions using transmission electron microscopy (TEM). Our data suggest that CTV residues, particularly K380, play an important role in ZapD mediated FtsZ lateral bundling. Furthermore, our data extend Buske and Levin’s results by revealing that the net-charge of the FtsZ CTV not only contributes to the lateral interaction potential of FtsZ but may also enhance longitudinal interactions within the FtsZ protofilament [22].

**Fig 3:**
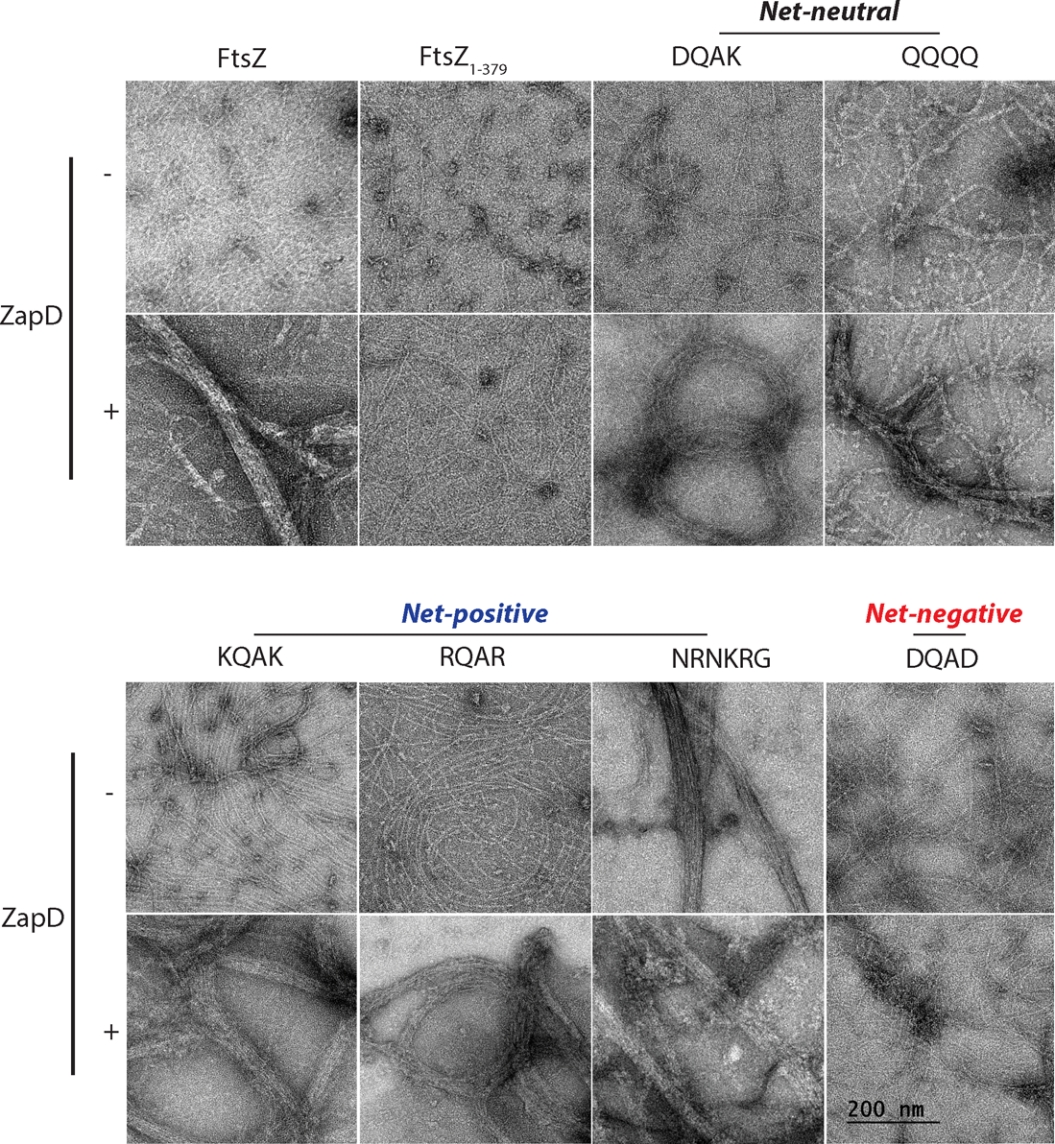
Morphologies of polymeric assemblies of FtsZ and FtsZ CTV mutant proteins. *In vitro* reactions containing FtsZ and FtsZ CTV mutants (5 ∝M) alone or combined with purified ZapD at 1:1 ratios in a polymerization buffer (50 mM K-MOPS pH 6.5, 50 mM KCl, 2.5 mM MgCl2, and 1 mM GTP) were incubated for 5 mins at room temperature. A 10-μl aliquot of each reaction was placed on carbon-coated copper grids (Electron Microscopy Sciences), processed and imaged as described in the material and methods section of the main text. Negative stained transmission electron microscopy images of FtsZ or FtsZ CTV mutants with or without ZapD are shown. Bar = 200 nm.

We observed that WT FtsZ shows typical single protofilaments in the presence of GTP and polymeric bundles upon addition of ZapD as previously reported in the literature (Fig 3) [25]. As expected, no FtsZ protofilaments were observed in the absence of GTP (data not shown). To visualize the FtsZ CTV mutants alone or in the presence of ZapD, we used equimolar ratios of ZapD and FtsZ, as the co-sedimentation profiles of ZapD at lower concentration (1 μM) were not significantly different than those at higher concentration (5 μM) (Fig 2). FtsZ missing the CTV sequences (FtsZ_1-379_) forms single protofilaments that are slightly shorter than WT FtsZ, confirming an earlier observation by Buske and Levin (Fig 3 and S3 Fig) [22]. Addition of ZapD fails to promote bundling of FtsZ_1-379_ polymers (Fig 3 and S3 Fig). The net-neutral DQAK mutant showed largely single protofilaments similar to WT FtsZ, while the QQQQ mutant displayed modest amounts of lateral bundling on its own, perhaps due to the tendency of poly-glutamine sequences to aggregate (Fig 3). This suggests that a net-neutral FtsZ CTV retains the ability to maintain a functional interaction with ZapD as in the presence of ZapD, both FtsZ variants showed significant increases in bundled forms similar to WT FtsZ (Fig 3). The net-negative DQAD mutant displayed single protofilaments in the presence of GTP and no detectable changes in polymerization were observed upon addition of ZapD (Fig 3).

An *E*. *coli* FtsZ CTV mutant with the net-positive *B*. *subtilis* NRNKRG is able to associate laterally to form filament bundles without the aid of modulatory proteins consistent with a previous report (Fig 3) [22]. However, other net-positive CTV mutants (KQAK and RQAR) form significantly longer protofilaments compared to WT FtsZ and only form polymeric assemblies such as rings or bundles in the presence of ZapD (Fig 3 and S3 Fig). These data show that a net-positive CTV impacts both longitudinal and lateral interactions of FtsZ assembly, likely depending on the length and residues of CTV, and that the CTV net-charge alone is not a primary determinant of FtsZ lateral interaction potential. This notion is reinforced by the observations that the disordered FtsZ linker domain, which is variable in length and amino acid content between species, is implicated in FtsZ lateral associations [17].

The *in vitro* assays indicate that in addition to the native KQAD CTV sequence, ZapD is also able to interact with net-neutral (DQAK and QQQQ) and net-positive (KQAK, RQAR, and NRNKRG) FtsZ variants but fails do so with one that lacks the CTV region or one carrying a net-negative DQAD sequence. These results suggest that ZapD primarily recognizes the FtsZ CTV through hydrogen bonding to the peptide backbone rather than specific amino acids. However, the data also suggest that a basic residue is preferred at position 380 of FtsZ to enable optimal interactions with ZapD since a K380D mutation leading to a DQAD sequence significantly reduces the FtsZ/ZapD interactions. This suggests that K380 likely participates in a charge-mediated interaction, perhaps through the formation of a salt-bridge with a negatively charged side chain on ZapD. The lysine in a sequence of reverse polarity (DQAK) can perhaps also contact an acidic residue of ZapD in that region due to the inherently flexible nature of the FtsZ CCTP [29,30]. That an electrostatic component contributes to the ZapD/FtsZ interactions is also suggested by a decrease in ZapD-mediated pelletable FtsZ amounts in physiological buffer conditions containing high salt (S4 Fig) While these data are consistent with *E*. *coli* FtsZ CTV residues, particularly K380, being an important interacting residue, it is highly likely that other conserved FtsZ CCTP residues belonging to the CTC region participate in establishing key contacts with ZapD. Furthermore, we cannot exclude the alternative possibility that the FtsZ CTV variants FtsZ_1-379_ and DQAD may alter the structure of the C-terminus in a manner that precludes ZapD from making critical contacts with the CTC region.

### Differential Viability of *ftsZ84(Ts)* Cells Expressing FtsZ CTV Variants *In Trans*

**Fig 4:**
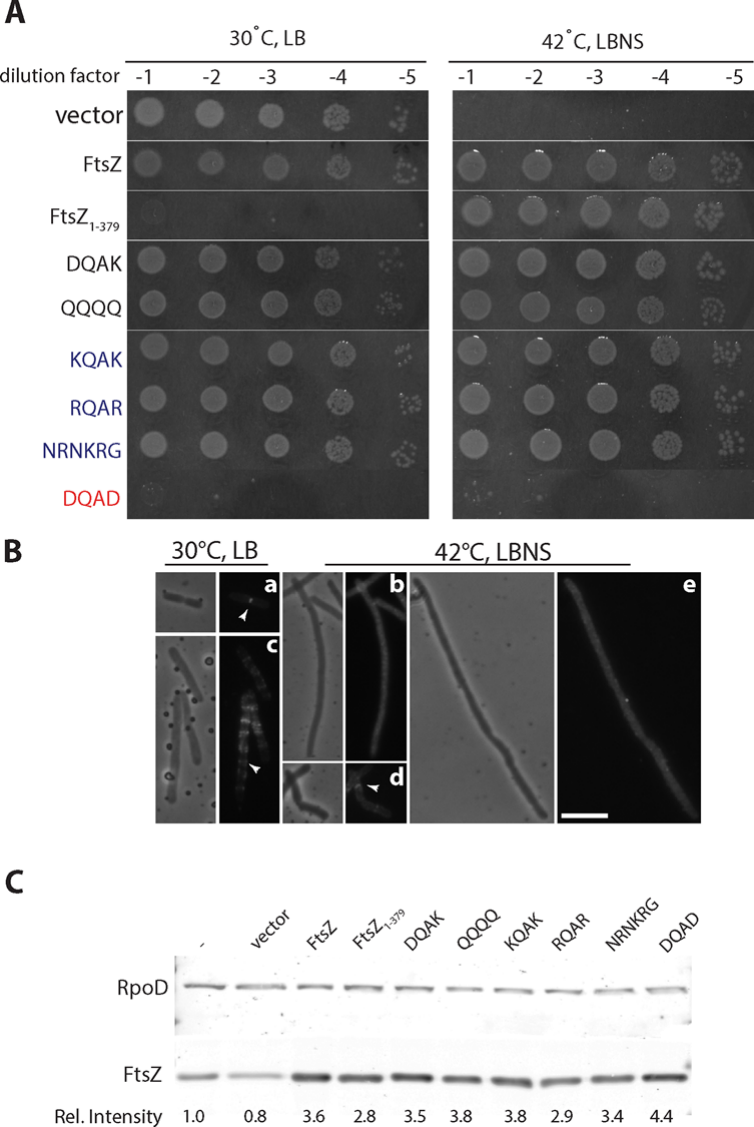
Spot-plate viabilities, Z-ring morphologies, and expression levels of FtsZ or FtsZ CTV mutants in *ftsZ84* (Ts) cells. **A.** FtsZ or FtsZ CTV mutants were maintained off of the low-copy pNG162 vector in the MGZ84 background carrying the *ftsZ84* (Ts) allele. Overnight cultures were normalized to OD_600_, serially diluted, and 3 μl aliquots were spotted on LB and LBNS agar plates with 1 mM IPTG plus appropriate antibiotics, and incubated at 30°C and 42°C as described in the material and methods section. At the permissive condition (30°C LB; left) FtsZ and FtsZ CTV mutants are able to support growth except FtsZ_1-379_ and DQAD. At the non-permissive condition (42°C LBNS; right) most FtsZ CTV mutants are able to support growth to WT levels except DQAD. **B.** FtsZ-ring morphologies as determined by immunofluorescence of MGZ84 cells expressing FtsZ_1-379_ or DQAD mutants *in trans* at mid-log phase (OD_600_ = ~0.6) during growth at permissive or restrictive conditions as described in the materials and methods section in the main text. **(a)** *ftsZ84* (Ts) cells grown at 30°C in LB; **(b)** *ftsZ84* (Ts) cells grown at 42°C in LBNS; **(c)** *ftsZ84* (Ts) cells with FtsZ_1-379_ expressed *in trans* grown at 30°C in LB; **(d)** *ftsZ84* (Ts) cells with FtsZ_1-379_ expressed *in trans* grown at 42°C in LBNS; and **(e)** *ftsZ84* (Ts) cells with DQAD expressed *in trans* grown at 42°C in LBNS. Both phase and fluorescence images are shown with arrowheads pointing to FtsZ-rings. Bar = 5 μm. **C.** Overnight cultures of MGZ84 strains bearing FtsZ and FtsZ CTV mutant plasmids were grown in permissive conditions and subcultured into LB at 30°C till OD_600_ = 0.2–0.3 at which point an aliquot was washed, and backdiluted to OD_600_ = 0.05 in LBNS media and transferred to 42°C. After one doubling (~25–30 mins) at 42°C, 1 mM IPTG was added and cells were grown for an additional two doublings (~1 hour). Cells were harvested for whole cell protein preparations and sampled at equivalent optical densities. Protein samples were analyzed by immunoblotting. RpoD was used as a loading and transfer control. ImageStudio software was used to quantify band intensities. Three independent experiments were conducted and a representative blot with relative intensities is shown.

FtsZ CTV mutant variants can impact FtsZ function *in vivo* given that K380, discussed above, is implicated in interactions with multiple FtsZ regulators in *E*. *coli*, and as shown here, with ZapD [24,26,27]. Therefore, we sought to examine the *in vivo* functionality of the FtsZ CTV mutants used in this study by examining the cell viability in an *ftsZ84* (Ts) background at the restrictive temperature. At 42°C, an *ftsZ84* (Ts) mutant fails to localize to the division site leading to lethal filamentation [54,55]. LB no salt (LBNS) media was used since it provides a more stringent condition for controlling the expression levels of *ftsZ84* (Ts) [56,57]. Our results indicate that most FtsZ CTV mutants examined here, including *E*. *coli* FtsZ with the *B*. *subtilis* NRNKRG CTV region (as shown previously; [22]), restore viability to *ftsZ84* (Ts) cells at the restrictive temperature (Fig 4). A net-negative CTV variant (DQAD), however, failed to complement *ftsZ84* (Ts) cells under both permissive and non-permissive conditions (Fig 4). Intriguingly, in addition to the DQAD variant, expression of FtsZ_1-379_ severely impaired growth of *ftsZ84* (Ts) cells only at the permissive condition (Fig 4). The reduced viability for some CTV variants and not others was not simply due to changes in stability or expression levels of plasmid-borne FtsZ CTV variants as they were all expressed within 2-fold of WT FtsZ levels *in trans* (Fig 4). Additionally, expression of FtsZ_1-379_ in the presence of WT FtsZ caused similar reduction in cell viability as in *ftsZ84* (Ts) cells at the permissive temperature (S5 Fig). The DQAD variant was dominant negative under both permissive and non-permissive conditions in the presence of either WT FtsZ or FtsZ84 (S5 Fig).

We further probed the reduced viability of FtsZ_1-379_ and the DQAD variant by visualizing the localization and morphologies of the Z-rings by immunofluorescence. Cells expressing the DQAD variant showed stable protein at levels similar to the other FtsZ variants expressed *in trans* (Fig 4). Yet Z-rings failed to localize in these cells under restrictive conditions suggesting that overexpression of DQAD perturbs the conformation of this variant protein such that it interferes with WT and FtsZ84 localization under both permissive and non-permissive conditions (Fig 4). FtsZ_1-379_ expressed *in trans* displayed aberrant Z-rings with significant filamentation under permissive conditions suggesting hyperstable assemblies of FtsZ84 (Fig 4). Conversely, under restrictive conditions, cells expressing the FtsZ_1-379_ variant displayed normal Z-rings without any significant filamentation (Fig 4). Furthermore, increasing salt concentrations at the restrictive temperature led to reduced viability of *ftsZ84* cells expressing FtsZ_1-379_, suggesting that rings forming under these conditions are hyperstabilized upon increased endogenous expression of *ftsZ84*, similar to what is observed under permissive conditions (S5 Fig).

It has been shown that an approximately 2-fold increase in expression of FtsZ84 can rescue the heat sensitivity of the *ftsZ84* (Ts) mutant [56]. Also it has been noted that *E*. *coli* FtsZ C-terminal tail mutants that fail to form rings in an *ftsZ* depletion strain can assemble rings in the *ftsZ84* (Ts) strain, leading to the suggestion that exogenous production of mutant FtsZ increases the concentration of the total FtsZ, and this in turn allows incorporation of both FtsZ84 and FtsZ mutants into a mixed ring [58]. The viability defects and Z-ring morphologies seen upon FtsZ_1-379_ overexpression at 30°C in LB, are consistent with endogenous FtsZ84 and FtsZ CTV variants co-assembling into hybrid ring assemblies that are defective in constriction. However, similar levels of FtsZ_1-379_ variant expression at 42°C in LBNS, lead to functional Z-ring assemblies with rings that are morphologically normal. These results suggest that Z-ring assemblies are mostly made up of exogenously expressed FtsZ under stringent *ftsZ* expression conditions (growth in LBNS) at the restrictive temperature. Furthermore, it is likely that the structural conformation of FtsZ_1-379_ is not significantly altered, as cells expressing the FtsZ_1-379_ variant under restrictive conditions are viable. However, our data do not rule out an alternative possibility that under more stringent conditions—high temperature and no salt, FtsZ84 polymers are unstable and these can counteract the hyperstabilizing effects of FtsZ_1-379_ but not the more severe effect of the DQAD variant. Expression of all the other FtsZ variants (DQAK, QQQQ, KQAK, RQAR, and NRNKRG) must not interfere with the essential activities of endogenous FtsZ84, as their overexpression does not affect the viability of *ftsZ84* (Ts) cells under permissive conditions.

### ZapD Fails to Localize to Midcell in the Absence of the FtsZ CTV Residues

ZapD binds FtsZ and accumulates at the midcell division site during the FtsZ-ring assembly and stabilization steps of *E*. *coli* cytokinesis, and aids in the efficiency of division. To assess the impact of FtsZ CTV mutants on both cell division and ZapD midcell localization *in vivo*, we examined both cell morphologies, and recruitment of a ZapD-GFP fusion to midcell in the presence of either WT FtsZ or various FtsZ CTV mutants expressed *in trans* in an *ftsZ84* (Ts) strain at 42°C. The DQAD mutant was not tested in these assays as it was dominant negative and failed to localize functional rings as described above. Our results reveal that cells expressing FtsZ_1-379_, NRNKRG, or DQAK variants showed a mix of filaments and normal length cells suggesting modest impairment of division. Additionally, ZapD-GFP failed to localize to midcell at appreciable frequencies in the absence of FtsZ CTV residues or in the presence of the NRNKRG variant. All other CTV variants, namely DQAK, QQQQ, KQAK, and RQAR, supported ZapD-GFP recruitment to midcell but only to moderate frequencies when compared to the WT (KQAD) sequence.

**Fig 5:**
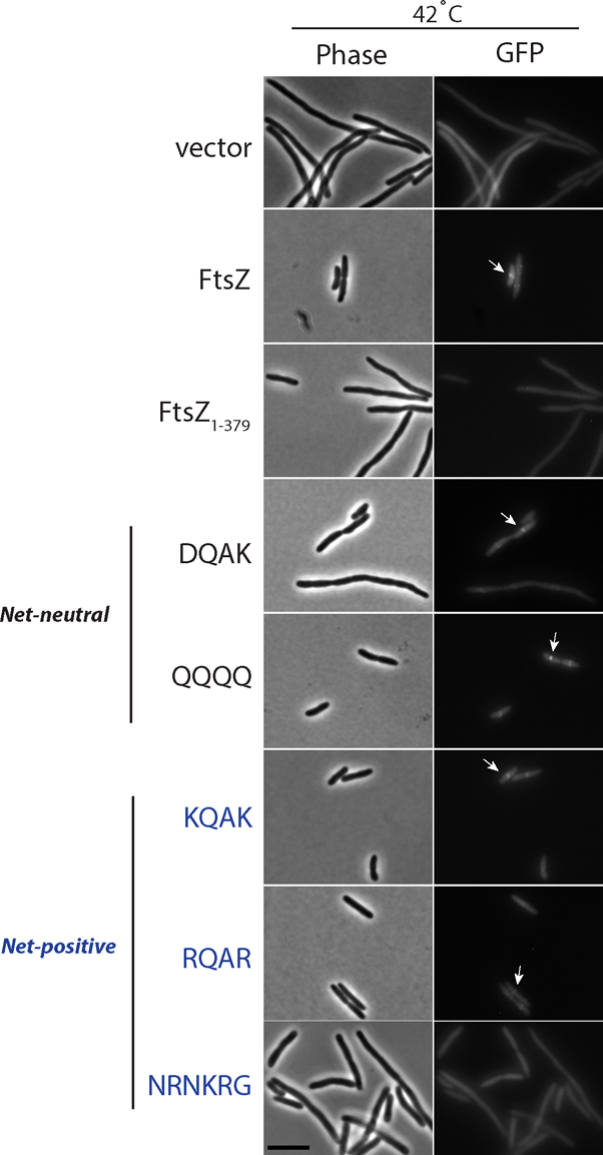
The FtsZ CTV region is required for localization of ZapD-GFP to midcell. Overnight cultures of AMZ84 cells expressing FtsZ or FtsZ CTV variants and a ZapD-GFP fusion *in trans* were subcultured in M63 glycerol minimal media in the presence of appropriate antibiotics at the permissive temperature (30°C) till OD_600_ = 0.2–0.3 at which point an aliquot was washed and backdiluted to OD_600_ = 0.05 in the same media and transferred to the restrictive temperature (42°C) for one doubling (~ 1 hour). Expression of FtsZ and ZapD were induced by addition of 1 mM IPTG and grown for an additional one-two doublings (~90 mins) at the same temperature. Fluorescent images were obtained as described in the materials and methods section. Arrows point to midcell ZapD-GFP fusion localization. Bar = 5 μm.

The cell lengths of the various FtsZ CTV mutants expressed *in trans* in an *ftsZ84* (Ts) background at 42°C displayed considerable variability suggesting that the CTV region has implications in supporting division in *ftsZ84 (Ts)* cells at the restrictive temperature (Table 4). At 42°C, the net-neutral QQQQ, and the net-positive KQAK and RQAR variants restore division similar to WT FtsZ levels (Fig 5 and Table 4). However, FtsZ_1-379_, a net-neutral (DQAK) mutant, or a NRNKRG variant, displayed a mix of filaments and WT cells (Fig 5 and Table 4). The plate viability of FtsZ_1-379_, DQAK, or NRNKRG variants showed no significant changes, although microscopic analysis reveals moderate degrees of division impairment confirming a prior report that expression of a plasmid-borne FtsZ_∆380–383_ lacking CTV residues in *ftsZ84* (Ts) cells show a modest reduction in viable plate counts at the restrictive temperature [27]. Since each FtsZ CTV variant was expressed to similar levels as WT FtsZ *in trans*, we attribute the division defects to not be simply a result of differential protein expression (S6 Fig). These data allude to the possibility of FtsZ_1-379_, DQAK, or a NRNKRG having altered structural conformation impacting interactions with multiple regulatory partners such as MinC, ClpX, SlmA, and ZapD, and influencing division (Fig 5 and Table 4).

**Table 4:** Cell lengths of strains expressing FtsZ or FtsZ CTV mutants *in trans* in *ftsZ84* (Ts) background.

Strain^a^	FtsZ/FtsZ CTV mutant^b^	30°C		42°C	
		Average ± SD (μm)^c^	N^d^	Average ± SD (μm)^c^	N^d^
Wild type	-	2.1 ± 0.6	394	2.8 ± 1.3	448
*ftsZ84*	-	2.4 ± 0.6	443	18.4 ± 17.4	391
	FtsZ	2.1 ± 0.5	365	3.4 ± 1.6	443
	FtsZ_1-379_	3.4 ± 1.2	434	7.1 ± 4.1	324
	FtsZ_DQAK_	2.4 ± 0.6	421	5.6 ± 4.3	369
	FtsZ_QQQQ_	2.1 ± 0.5	344	3.2 ± 1.3	399
	FtsZ_KQAK_	1.8 ± 0.4	458	3.4 ± 1.6	449
	FtsZ_RQAR_	2.0 ± 0.4	435	2.9 ± 1.5	508
	FtsZ_NRNKRG_	2.0 ± 0.6	497	4.3 ± 2.6	439

^a^ Strains backgrounds were TB28 and AMZ84.

^b^ Plasmids pNG162 bearing FtsZ and FtsZ CTV mutants and pDSW208 carrying a ZapD-GFP fusion were expressed *in trans* and grown in M63 glycerol minimal medium at 30°C, till OD_600_ = 0.2–0.3 at which point an aliquot was washed and transferred to 42°C in the same media. After one doubling (~1 hour) at 42°C, 1 mM IPTG was added and cells were grown for an additional one-two doublings (~75–90 mins) at which point cells were imaged. Cells were also imaged at 30°C prior to transfer to 42°C. Cell lengths were measured as described in the text.

^c^ SD = Standard Deviation.

^d^ N represents the number of individual cells measured for each strain. In case of filamentous strains, fewer numbers of cells were present within each field of view.

We first confirmed that a ZapD-GFP fusion protein localizes to midcell at 42°C in *ftsZ84* (Ts) cells when WT FtsZ is expressed *in trans*. The fusion protein however failed to display appreciable amounts of localization in the presence of FtsZ_1-379_ indicating an important role for CTV residues in recruiting ZapD to midcell (Fig 5 and Table 5). Loss of ZapD localization in these cells is not due to lack of localization of FtsZ_1-379_ as immunofluorescence images revealed normal Z-rings under these conditions (Fig 4). These data are also consistent with a prior study where GFP fused to FtsZ_∆380–383_ displayed normal Z-rings [27]. Cells expressing the net-positive *B*. *subtilis* CTV (NRNKRG) mutant also failed to accumulate ZapD-GFP at appreciable frequencies at midcell (Fig 5 and Table 5). Although ZapD was able to bind the FtsZ NRNKRG variant *in vitro*, it was not able to enhance sedimentable amounts of the NRNKRG mutant. Together these results suggest that the ZapD/NRNKRG binding can either be better accommodated *in vitro* or is non-specific. It is also conceivable that some other FtsZ regulator binds the NRNKRG variant with greater affinity in the cell thereby outcompeting ZapD localization to midcell. Net-neutral (DQAK and QQQQ) and other net-positive CTV mutants (KQAK and RQAR) all support localization of ZapD-GFP to midcell although ZapD-GFP localization frequencies were not as robust as those in cells expressing WT KQAD residues (Fig 5 and Table 5). Differences in ZapD-GFP localization frequencies were not simply a result of changes in protein levels, due to plasmid-borne expression of FtsZ or ZapD, since all FtsZ CTV variants were expressed to similar levels as WT FtsZ *in trans*, and ZapD-GFP levels were also similar amongst the various strains (S6 Fig). However, we cannot exclude the possibility that ZapD recruitment may be sensitive to differences in polymer stability or bundling of the FtsZ variants in the cell.

**Table 5:** Midcell localization frequencies of a ZapD-GFP fusion in *ftsZ84* cells grown under restrictive conditions.

Strain background^a^	FtsZ/FtsZ CTV mutant^b^	42°C	
		Average ± SD (%)^c^	N^d^	
*ftsZ84*	vector	ND	410	
	FtsZ	65.1 ± 11.8	679	
	FtsZ_1-379_	1.1 ± 1.2	421	
	FtsZ_DQAK_	37.8 ± 7.2	362	
	FtsZ_QQQQ_	33.3 ± 4.7	877	
	FtsZ_KQAK_	37.2 ± 5.0	560	
	FtsZ_RQAR_	21.6 ± 6.9	830	
	FtsZ_NRNKRG_	ND	620	

^a^ Strains background was AMZ84.

^b^ Plasmids pNG162 bearing FtsZ and FtsZ CTV mutants and pDSW208 carrying a ZapD-GFP fusion were expressed *in trans* and grown in minimal M63 glycerol medium at 30°C, till OD_600_ = 0.2–0.3 at which point an aliquot was washed and transferred to 42°C in the same media. After one doubling (~1 hour) at 42°C, 1 mM IPTG was added and cells were grown for an additional one-two doublings (~75–90 mins) at which point cells were imaged.

^c^ Midcell localization of ZapD-GFP was quantified using ImageJ and is reported as average ± standard deviation (SD) of three different colonies. ND = not detectable.

^d^ N represents the total number of individual cells measured for each strain from three different colonies. In case of filamentous strains, fewer numbers of cells were present within each field of view.

Taken together, the *in vivo* results underscore the importance of CTV residues in mediating interactions with ZapD. While K380 in *E*. *coli* is a critical interacting residue with multiple FtsZ regulatory proteins, MinC, SlmA, and ClpX, this study and other work indicate that removal of K380 in the context of removing the entire CTV, or substitutions with Q (this study), A or M are largely tolerated by the cell [24,26,27]. This indicates that the lysine is not absolutely necessary but that it plays a critical role, perhaps by forming a salt-bridge with an acidic residue in ZapD and in facilitating the structural conformation of all the CCTP residues that contact ZapD.

### Most β- and γ-Proteobacterial Species with Net-Neutral FtsZ CTV Regions Retain a *zapD* Homolog

A preliminary phylogenetic analysis of proteobacterial classes revealed that orthologs of *zapD* are restricted to the β- and γ-proteobacteria [25]. To explore the possibility that ZapD was restricted to bacterial phyla where the FtsZ CTV sequences were similar to those of *E*. *coli* FtsZ CTV, we analyzed FtsZ C-terminal tail sequences from 427 select α-, β- and γ-proteobacterial species with fully sequenced genomes and without ambiguities in their taxonomic classification. Of these, ~88% γ-species and ~94% of β-species have a net-neutral FtsZ CTV while an overwhelming majority (≥ 99%) of the α-proteobacterial species contained net-positive CTV amino acid content. Orthologs of *zapD* were present only in β- and γ-species and were predominantly associated with net-neutral FtsZ CTV sequences (Table 6). Strikingly, only ~5% of the species analyzed contained net-negative FtsZ CTV sequences and these were present only in the γ-proteobacterial class. Of the 277 β- and γ-species analyzed, a majority (~86%) contained net-neutral (KQAD, RQAD or KQAD-like) amino acids in their FtsZ CTV and of these a majority (~98%) retain a *zapD* homolog in their genomes (Table 6). These results indicate a correlation between the presence of a net-neutral FtsZ CTV and presence of ZapD. While it’s not surprising that species closely related to *E*. *coli* have similar FtsZ CTV residues and retain ZapD, a possible interpretation is that such a CTV region plays a structural role in defining the ZapD binding site in these species. It is also tempting to speculate that the role of ZapD in bacterial species that contain KQAD-like and/or mostly net-neutral FtsZ CTV sequences may in part serve to enhance the lateral interaction potential of FtsZ. Refer to S2 Table for the entire list of species analyzed.

**Table 6:** Phylogenetic analysis of the FtsZ CTV region correlated to the presence of *zapD* orthologs in proteobacteria.^a^

**A. Presence of *zapD* orthologs in relation to the net-charge of FtsZ CTV sequences.**											
**Class**	***zapD***	**Net-Charge of FtsZ CTV sequence**									
		**Neutral**				**Positive**			**Negative**		
α	‒	1				149			0		
β	+	72				2			0		
	‒	0				3			0		
γ	+	109				1			4		
	‒	66				15			5		
**B. Presence of *zapD* orthologs and nature of FtsZ CTV amino acid residues.**											
**Class**	***zapD***	**FtsZ CTV Sequence**									
		**KQAD-like**				**Non-KQAD-like**					
		**KQAD**	**RQAD**	**Other**	**Total**	**RQNN**	**RQEE/A**	**VPSN**	**KKVK**	**Other**	**Total**
β	+	66	5	0	71	2	0	1	0	0	3
	‒	0	0	0	0	0	0	0	0	3	3
γ	+	80	27	3	110	0	3	0	1	0	4
	‒	6	41	9	56	0	1	0	0	29	30

^a^ One hundred fifty alpha-, 77 beta-, and 247 gamma-proteobacterial species were analyzed.

## Conclusions

In the present study we sought to define the interaction of the *E*. *coli* FtsZ regulatory protein ZapD, with the FtsZ C-terminal variable (CTV) region. Our results provide insights into two aspects of FtsZ assembly in *E*. *coli* and related bacteria: (i) the role of the FtsZ CTV residues in the interaction with ZapD, and (ii) the role of the FtsZ CTV on FtsZ-ring assembly and cytokinesis. Our data support a model in which the FtsZ CTV residues provide an optimal structural conformation that allows ZapD to interact with the FtsZ CCTP. Although a lysine at position 380 is not absolutely required *in vivo*, it appears to contribute a critical electrostatic interaction since ZapD binding to FtsZ is significantly decreased by a K380D substitution *in vitro*. The ZapD/FtsZ interactions are somewhat reminiscent of what is seen with ZipA, and the *B*. *subtilis* FtsZ regulator SepF, interactions with FtsZ CCTP [23,29,59]. The ZipA/FtsZ CCTP interaction is predominantly through hydrophobic interactions and hydrogen bond formation with the peptide backbone [19,23,29]. The interaction of SepF with FtsZ CCTP is also deemed to be largely through hydrophobic contacts and recognition of secondary and tertiary structural conformations, rather than specific amino acids [59]. However, changes in the FtsZ CTV residues, do reduce the SepF/FtsZ interaction suggesting that CTV residues may provide some additional electrostatic interactions [59]. The FtsZ CTV is also involved in *T*. *maritima* FtsA/CCTP interaction through the formation of a salt bridge with the extreme C-terminal amino acid residue [30]. Collectively, the data suggest that the CCTP, found at the end of the disordered linker, is driven towards specific structural conformations in the presence of specific binding partners. It has been shown that intrinsically disordered regions (IDR) and intrinsically disordered proteins (IDP), common in eukaryotic cells, have evolved this property to engage multiple binding partners [19,60,61]. We propose that the ZapD/FtsZ interaction drives the conformation of the FtsZ CCTP in a similar manner and that the CTV residues play non-specific roles, such as steering or charge stabilization, in this process.

In addition to a role for FtsZ CTV in the interactions with ZapD, our work extends previous observations that the net-charge of CTV contributes to not only the lateral interaction potential but perhaps also the longitudinal associations of FtsZ protofilaments *in vitro*. While the CTV residues from *B*. *subtilis* lead to enhanced lateral interactions of *E*. *coli* FtsZ protofilaments *in vitro*, they do not support robust division in *E*. *coli* suggesting that *B*. *subtilis* CTV residues cannot provide the optimal structural definition for interactions with the various binding partners present in *E*. *coli*. Chimeric *B*. *subtilis* FtsZ with *E*. *coli* CTV residues do not support division in *B*. *subtilis* and it was suggested that this maybe due to the loss of lateral interaction potential [22]. But *B*. *subtilis* FtsZ linker mutants, that don’t support FtsZ lateral interaction potential, are still viable for division [17]. These data suggest that in the cell, in addition to their role in FtsZ assembly dynamics, the FtsZ CTV region provides a stabilizing structural motif that aids in interactions of multiple binding partners with the FtsZ CCTP. More detailed information of the precise role(s) of CTV residues in Z-ring assembly and interactions with FtsZ-regulatory proteins can be obtained from studying FtsZ CTV variants in the chromosomal context, and by employing structure-function studies of the FtsZ CCTP with different binding partners, including ZapD.

## Supporting Information

S1 Fig Immunoblot of FtsZ, FtsZ CTV mutants, and ZapD levels in yeast. (PDF)

S2 Fig Sedimentation of ZapD with FtsZ NRNKRG variant at different ratios of the two proteins. (PDF)

S3 Fig Electron micrographs and protofilament lengths of FtsZ, FtsZ_1-379_, KQAK, and RQAR mutants. (PDF)

S4 Fig Sedimentation of ZapD with FtsZ under physiological conditions. (PDF)

S5 Fig Plating viability, and relative expression levels of FtsZ and FtsZ CTV mutants in *ftsZ84* (Ts) cells. (PDF)

S6 Fig Relative expression levels of FtsZ, FtsZ CTV mutants, and ZapD-GFP in *ftsZ84* (Ts) cells. (PDF)

S1 Table List of primers used in the study. (PDF)

S2 Table Complete list of α-, β- and γ-proteobacterial species analyzed in this study. FtsZ CCTP sequences and the correspondence of *zapD* orthologs are reported. The *zapD* orthologs are listed based on % ORF coverage, % identity, and % similarity (positive).
(XLSX)

S1 Text Supplementary Data. (PDF)
