# Supplementary figures and images for: Characterization of the FtsZ C-Terminal Variable (CTV) Region in Z-Ring Assembly and Interaction with the Z-Ring Stabilizer ZapD in E. coli Cytokinesis
Source: PLoS One. 2016 Apr 18;11(4):e0153337. doi: 10.1371/journal.pone.0153337 (PMC4835091; doi:10.1371/journal.pone.0153337)

S1 Fig.

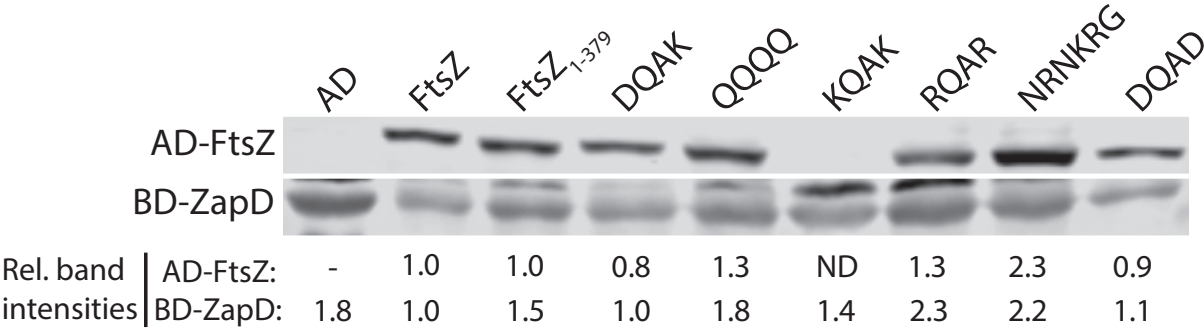

Supplement: S1 Fig — (PDF) [file pone.0153337.s001.pdf]

S2 Fig.

A

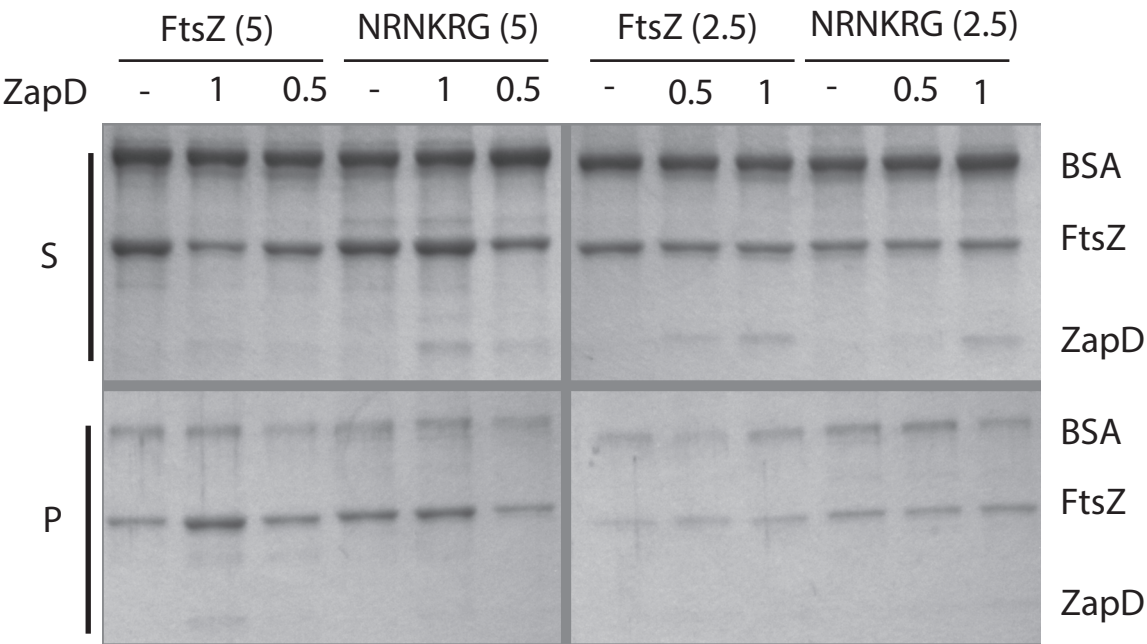

B

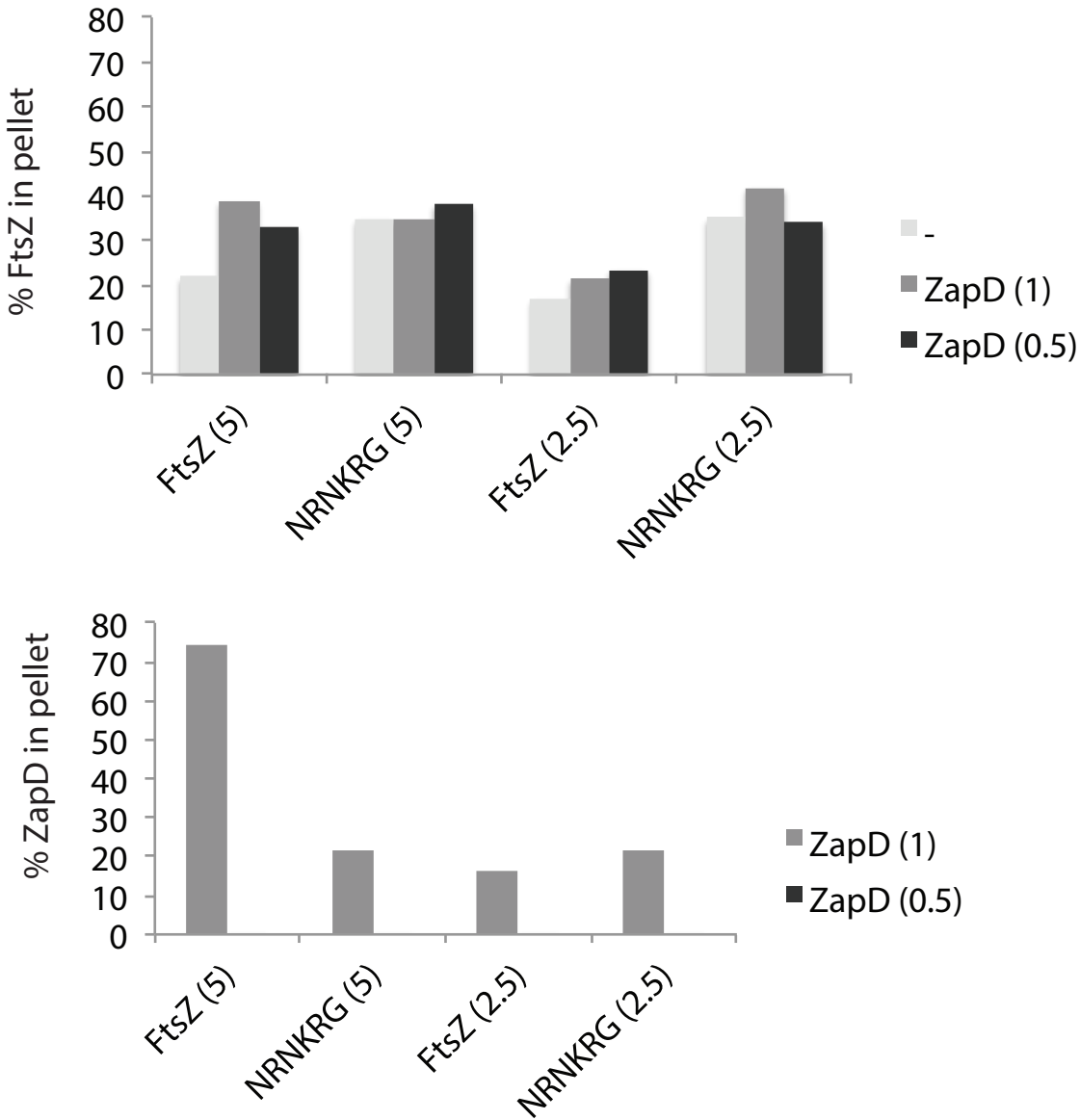

Supplement: S2 Fig — (PDF) [file pone.0153337.s002.pdf]

S3 Fig.

A

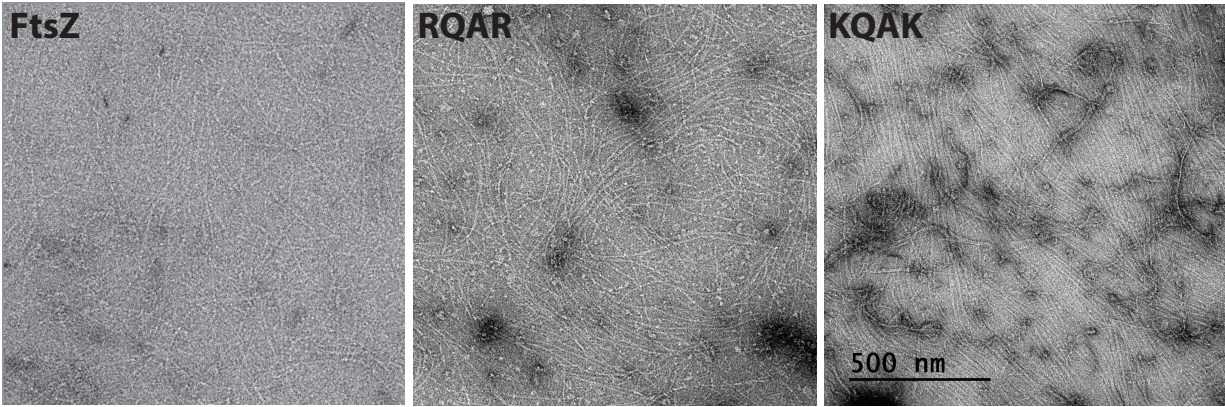

B

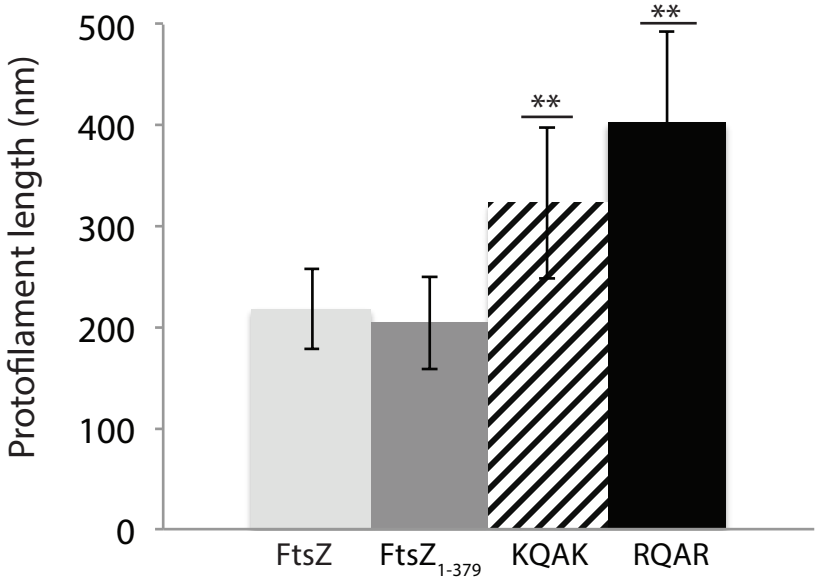

C

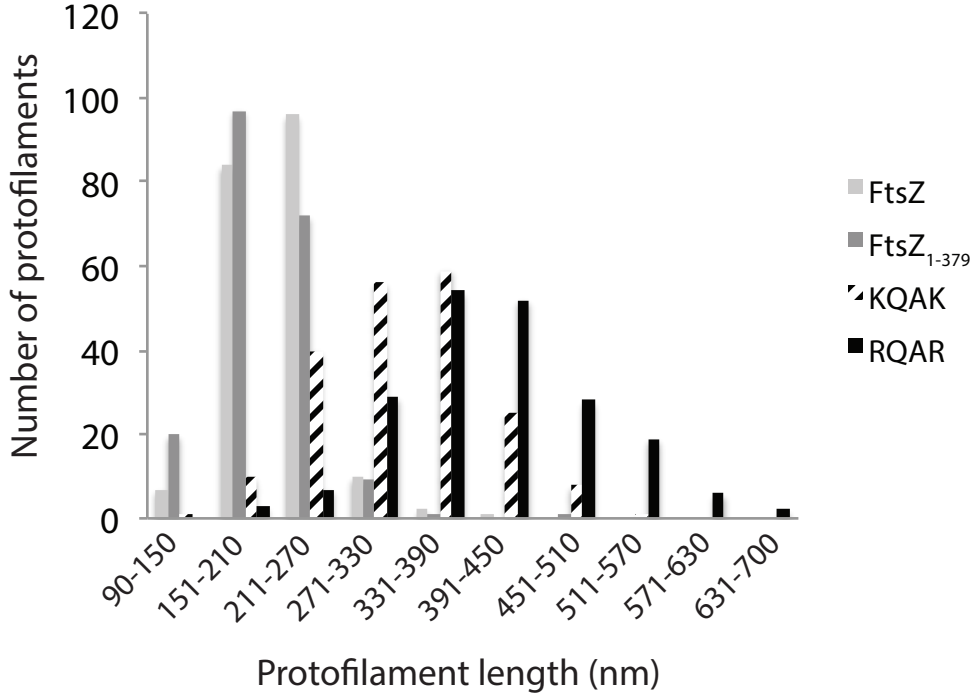

Supplement: S3 Fig — (PDF) [file pone.0153337.s003.pdf]

S4 Fig.

A

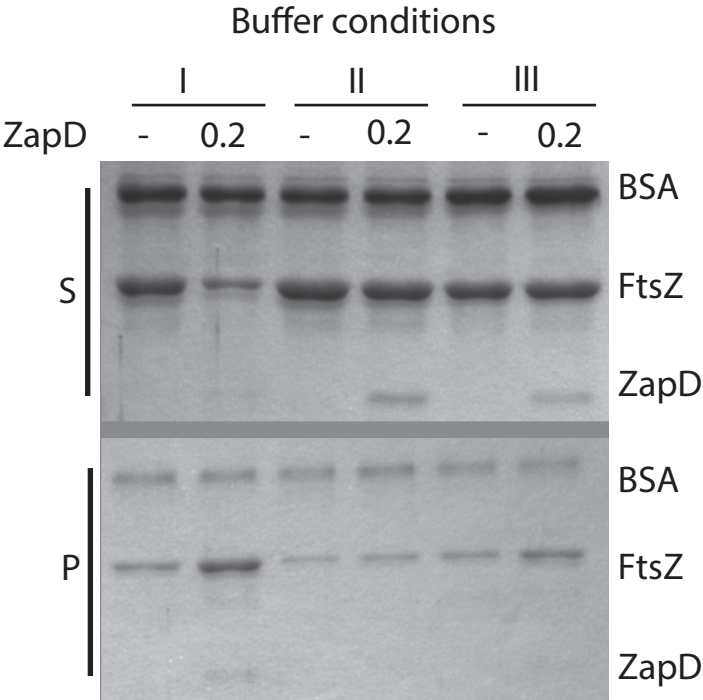

B

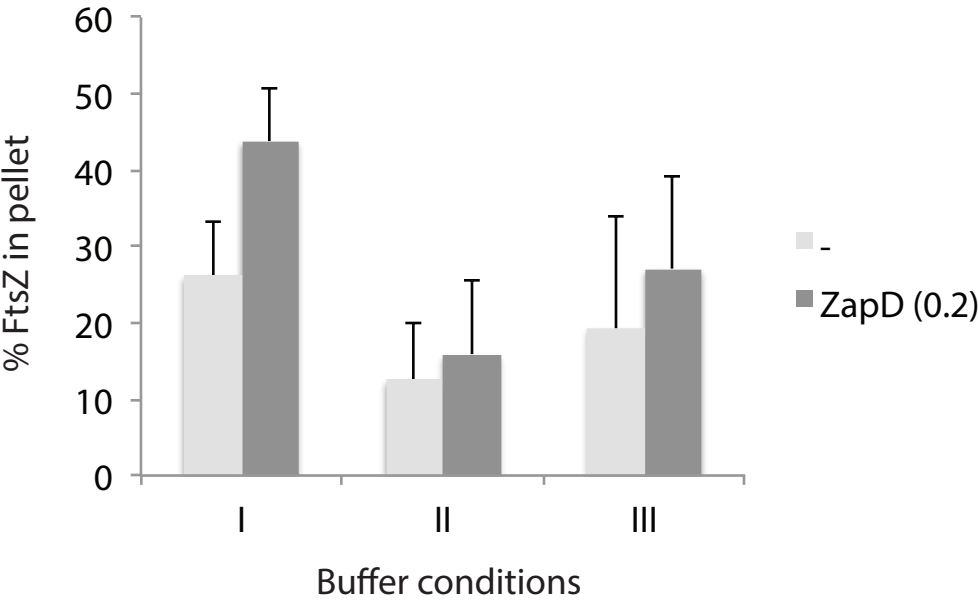

Supplement: S4 Fig — (PDF) [file pone.0153337.s004.pdf]

S5 Fig.

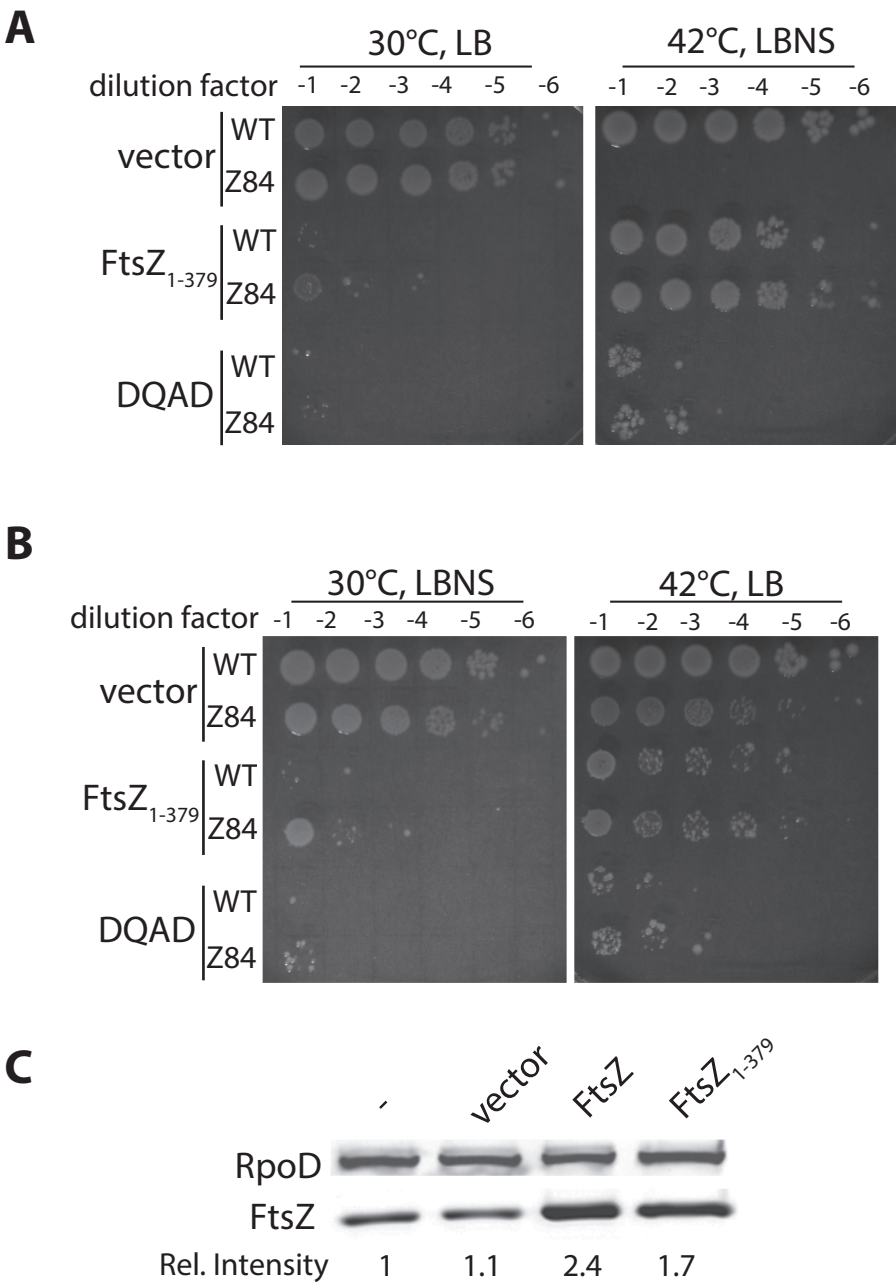

Supplement: S5 Fig — (PDF) [file pone.0153337.s005.pdf]

S6 Fig.

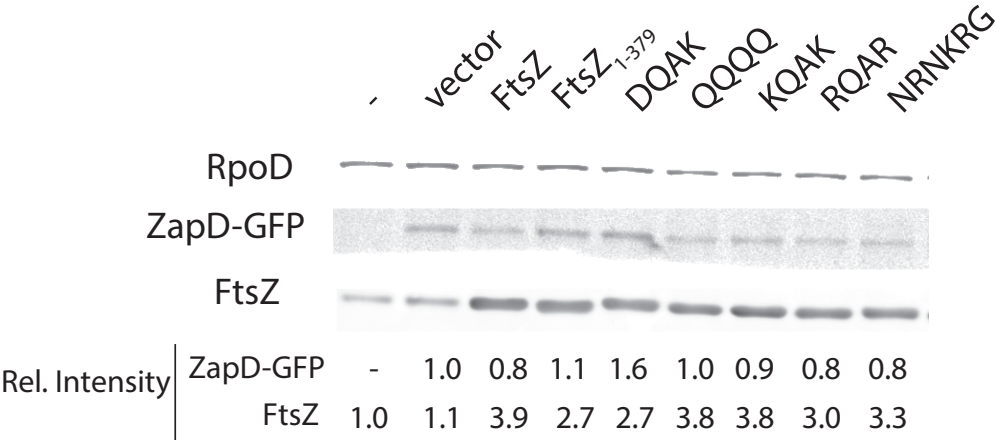

Supplement: S6 Fig — (PDF) [file pone.0153337.s006.pdf]
