# Supplementary material for: Characterization of the FtsZ C-Terminal Variable (CTV) Region in Z-Ring Assembly and Interaction with the Z-Ring Stabilizer ZapD in E. coli Cytokinesis
Source: PLoS One. 2016 Apr 18;11(4):e0153337. doi: 10.1371/journal.pone.0153337 (PMC4835091; doi:10.1371/journal.pone.0153337)
Supplement: S1 Table — (PDF) [file pone.0153337.s007.pdf]

S1 Table. List of primers used in the study.

Name	Sequence (5′-3′)	Site	Plasmid
FtsZFwd-GW	GGGGACAACTTTGTACAAAAAAGTTGGCATGTTTGAACCAA		pKHH1-6 pKHH17,18
FtsZ379Rev-GW	GGGGACAACTTTGTACAAGAAAGTTGGTTAACGCAGGAATGCTGGGATATCCAG		pKHH1
FtsZ383Rev$_{K380D}$-GW	GGGGACAACTTTGTACAAGAAAGTTGGTTAATCAGCTTGATCACGCAGGAA		pKHH2
FtsZ383Rev$_{D383K}$-GW	GGGGACAACTTTGTACAAGAAAGTTGGTTACTTAGCTTGCTTACGCAGGAA		pKHH3
FtsZ383Rev$_{K380D\ D383K}$-GW	GGGGACAACTTTGTACAAGAAAGTTGGTTACTTAGCTTGATCACGCAGGAATGCTGGGATATC		pKHH4
FtsZ383Rev$_{QQQQ}$-GW	GGGGACAACTTTGTACAAGAAAGTTGGTTATTGTTGTTGTTGACGCAGGAATGCTGGGATATC		pKHH5
FtsZ383Rev$_{RQAR}$-GW	GGGGACAACTTTGTACAAGAAAGTTGGTTAACGAGCTTGACGACGCAGGAATGC		pKHH17
FtsZ383Rev$_{NRNKRG}$-GW	GGGGACAACTTTGTACAAGAAAGTTGGTTAGCCGCGTTTATTACGGTTACG		pKHH18
ZapD Fwd-pBad33	CGCGCGAGCTCAGGAGGAAGGCGATGCAGACCCAGGTCCTTTTTGA	SacI	pKHH19
AJ yacF Rev-nonstop	AAAAGTCGACGCAACAGGCCAGTTCGAA	SalI	pKHH19
FtsZ1Fwd-pNG162	CGCGCCCATGGAGGAGGAAGGCGATGTTTGAACCAATGGAA	NcoI	pAM1-2, pKHH11-17
FtsZ383Rev-pNG162	GCGCGCAGGCTTTTAATCAGCTTGCTTACG	HindIII	pKHH11
FtsZ379Rev-pNG162	GCGCGCAAGCTTTTAACGCAGGAATGCTGGGATATCCAG	HindIII	pKHH12
FtsZ383Rev$_{K380D}$-pNG162	GCGCGCAAGCTTTTAATCAGCTTGATCACGCAGGAA	HindIII	pKHH13

FtsZ383Rev$_{D383K}$-pNG162	GCGCGCAAGCTTTTACTTAGCTTGCTTACGCAGGAA	HindIII	pKHH14
FtsZ383Rev$_{K380D D383K}$-pNG162	GCGCGCAAGCTTTTACTTAGCTTGATCACGCAGGAATGC	HindIII	pKHH15
FtsZ383Rev$_{QQQQ}$-pNG162	GCGCGCAAGCTTTTATTGTTGTTGTTGACGCAGGAATGCTGGGAT	HindIII	pKHH16, pLT1
FtsZ383Rev$_{K380R D383R}$-pNG162	GCGCGCAAGCTTTTAACGAGCTTGCTTAGCCAGGAATGC	HindIII	pAM1
FtsZ$_{CTVB}$Rev-pNG162	GGCGGTCGACTTAGCCGCGTTTATTACGGTTACG	SalI	pAM2, pAM3
FtsZ1Fwd-pET21b	GATCGATCCATATGTTTGAACCAATGGAACTT	NdeI	pAM3, pKHH7-10, pLT1
FtsZ383Rev$_{K380D}$-pET21b	GATCGGATCCTTAATCAGCTTGATCACGCAGGAATGCTGGGAT	BamHI	pKHH7
FtsZ383Rev$_{D383K}$-pET21b	GATCGGATCCTTACTTAGCTTGCTTACGCAGGAATGC	BamHI	pKHH8
FtsZ383Rev$_{K380R D383R}$-pET21b	GATCGGATCCTTACTTAGCTTGATCACGCAGGAATGCTGGGAT	BamHI	pKHH9
