# Supplementary material for: Characterization of the FtsZ C-Terminal Variable (CTV) Region in Z-Ring Assembly and Interaction with the Z-Ring Stabilizer ZapD in E. coli Cytokinesis
Source: PLoS One. 2016 Apr 18;11(4):e0153337. doi: 10.1371/journal.pone.0153337 (PMC4835091; doi:10.1371/journal.pone.0153337)
Supplement: S1 Text — (PDF) [file pone.0153337.s009.pdf]

Supplementary Data

Figure Legends

S1 Fig. Immunoblot of FtsZ, FtsZ CTV mutants, and ZapD levels in yeast. Cell lysates from diploid yeast cells co-expressing AD (activation domain) fused to FtsZ or FtsZ CTV mutants, and BD (DNA binding domain) fused to ZapD, were obtained by repeated freeze-thaw cycles and sonication (1). Cell volumes harvested were normalized to optical densities, and equal aliquots were separated on a 10% SDS-PAGE gel. Immunoblotting was performed using an anti-AD rabbit polyclonal (Sigma, 1:500) and a peptide derived anti-ZapD rabbit polyclonal (Genscript, 1:1000). An appropriate IRDye secondary antibody (1:20,000) was used for detection using an Odyssey CLx Infrared imaging system (LI-COR). Band intensities were quantified using ImageStudio software (LI-COR). A representative blot with relative band intensities is shown.

S2 Fig. Sedimentation of ZapD with FtsZ NRNKRG variant at different ratios of the two proteins.

A. FtsZ and NRNKRG mutants (5 μM or 2.5 μM) were incubated alone or combined with purified ZapD (1 μM or 0.5 μM) at different ratios in a polymerization buffer (50 mM K-MOPS pH 6.5, 50 mM KCl, 2.5 mM $MgCl_2$, and 1 mM GTP) containing 3 μM BSA. Reactions were processed as outlined in the main text. Note, concentrations of proteins in each reaction are shown in parenthesis. Equivalent aliquots (5 μls) of pellet (bottom panel) and supernatants (top panel) were resolved on SDS-PAGE, stained with SimplyBlue SafeStain, and imaged.

B. Quantification of the percent of FtsZ, NRNKRG, and ZapD present in the pellet in each reaction containing different concentrations of the two proteins is shown. In reactions without

ZapD, compared to WT FtsZ, more pelletable amounts of the NRNKRG variant were recovered even at low concentrations of the protein. In reactions containing 1 μM ZapD with 5 μM of FtsZ, increases in pelletable FtsZ are noted with ~75% ZapD co-sedimenting in the pellet. In contrast, ~20% of ZapD is present in the pellet in reactions containing NRNKRG but there is no significant increase in the pelletable amounts of NRNKRG. This is also the case in reactions containing 0.5 μM ZapD and lower amounts of NRNKRG. The lower concentrations of ZapD (0.5 μM) could not be detected in the pellet fraction under the assay conditions, although the amount of pelletable WT FtsZ increased 1.5X in reactions containing 5 μM FtsZ. A representative image with quantification of three independent experiments is shown.

S3 Fig. Electron micrographs and protofilament lengths of FtsZ, $FtsZ_{1-379}$, KQAK, and RQAR mutants.

A. Negative stained transmission electron microscopy images of FtsZ, RQAR, and KQAK protofilament morphologies are shown at a magnification of X 15,000 or X 20,000 to enable better visualization of protofilament lengths. Bar = 500 nm.

B. The average protofilament length of FtsZ ± standard deviation (217.7 ± 39.2 nm) was compared to $FtsZ_{1-379}$ (204.2 ± 45.5 nm), KQAK (322.8 ± 74.3 nm) and RQAR (402.8 ± 89.6 nm) using a one-way ANOVA analysis ($\alpha = 0.001$). FtsZ lacking CTV residues ($FtsZ_{1-379}$) filaments were slightly shorter compared to WT FtsZ, and net-positive FtsZ CTV mutants KQAK and RQAR were significantly longer ($p < 0.001$; **) compared to WT FtsZ protofilaments. Two hundred protofilaments were measured per FtsZ or FtsZ variant in two independent trails. Error bars represent the standard deviation from the mean.

C. The length distribution of FtsZ and FtsZ CTV mutant protofilaments (5 μM) measured from

electron micrograph images. The reactions were conducted and imaged as described in the main text. Two hundred protofilaments from two independent trials were measured per FtsZ.

S4. Fig. Sedimentation of ZapD with FtsZ under physiological conditions.

A. FtsZ (5 μM) was incubated alone or combined with purified ZapD at 1:0.2 ratio in the following polymerization buffers (I: 50 mM K-MOPS pH 6.5, 50 mM KCl, 2.5 mM $MgCl_2$; II: 50 mM HEPES-NaOH pH 7.5, 200 mM KCl, 2.5 mM $MgCl_2$; III: 50 mM HEPES-NaOH pH 7.5, 50 mM KCl, 10 mM $MgCl_2$). All reactions contained 1 mM GTP and 3 μM BSA. Reactions were processed as outlined in the main text. Equivalent aliquots (5 μl) of pellet (bottom panel) and supernatants (top panel) were resolved on SDS-PAGE, stained with SimplyBlue SafeStain, and imaged. A representative image of two independent experiments is shown.

B. Quantification of the percent of FtsZ present in the pellet with or without ZapD in each reaction condition is shown. Buffer compositions are as described above. Under physiological conditions (II), low amounts of pelletable FtsZ is recovered. Addition of ZapD does not promote significant increases in sedimentable FtsZ amounts under these conditions. However, when concentration of Mg++ ions is increased in the buffer (III), increased levels of ZapD-mediated sedimentable FtsZ is noted. However, these levels are lower that those seen in standard buffer conditions (I).

S5 Fig. Plating viability, and relative expression levels of FtsZ and FtsZ CTV mutants in *ftsZ84* (Ts) cells.

A. FtsZ, $FtsZ_{1\text{-}379}$, and DQAD mutants were maintained off of the low-copy pNG162 vector in the MG1655 or MGZ84 background carrying the *ftsZ84* (Ts) allele. Overnight cultures were

normalized to OD_{600} = 1, serially diluted, and 3 μl aliquots were spotted on LB and LBNS agar plates with 1 mM IPTG plus appropriate antibiotics, and incubated at 30 °C and 42 °C as described in the main text. At the permissive condition (30°C LB; left) only WT FtsZ is able to support growth. At the non-permissive condition (42°C LBNS; right) $FtsZ_{1\text{-}379}$ mutant is able to support growth to WT levels. The DQAD mutant is dominant negative under the growth conditions assayed.

B. FtsZ, $FtsZ_{1\text{-}379}$, and DQAD mutants were maintained off of the low-copy pNG162 vector in the MG1655 or MGZ84 background carrying the *ftsZ84* (Ts) allele. Overnight cultures were normalized to OD_{600} = 1, serially diluted and 3 μl aliquots were spotted on LB and LBNS agar plates with 1 mM IPTG plus appropriate antibiotics, and incubated at 30 °C and 42 °C as described in the main text. Control plates grown in LBNS at the permissive temperature (30 °C; left), and grown in LB at the restrictive temperature (42 °C; right) are shown. $FtsZ_{1\text{-}379}$ now supports only partial viability of *ftsZ84* (Ts) cells at the restrictive temperature, but DQAD is still able to support very little growth under the assay conditions.

C. Overnight cultures of MGZ84 cells bearing FtsZ and $FtsZ_{1\text{-}379}$ mutant plasmids were grown in permissive conditions and subcultured in LB at 30 °C till OD_{600} = ~0.2 at which point 1 mM IPTG was added and cells were grown for an additional two doublings (~70 mins). Cells were harvested for whole cell protein preparations and sampled at equivalent optical densities. Protein samples were analyzed by immunoblotting. RpoD was used as a loading and transfer control. ImageStudio (LI-COR) software was used to quantify band intensities. The experiment was repeated three times and a representative blot with relative band intensities of FtsZ is shown.

S6 Fig. Relative expression levels of FtsZ, FtsZ CTV mutants, and ZapD-GFP in *ftsZ84* (Ts)

cells.

Plasmids pNG162 bearing FtsZ or FtsZ CTV mutants, and pDSW208 carrying a ZapD-GFP fusion were expressed *in trans* in AMZ84 cells and grown in M63 glycerol minimal medium at 30 °C, till OD_{600} = 0.2 - 0.3 at which point an aliquot was washed, backdiluted in the same media to OD_{600} = 0.05 and transferred to 42 °C. After one doubling (~1 hour) at 42 °C, 1 mM IPTG was added and cells were grown for an additional one-two doublings (~75-90 mins). Cells were harvested for whole cell protein preparations and sampled at equivalent optical densities. Protein samples were analyzed by immunoblotting. RpoD was used as a loading and transfer control. ImageStudio (LI-COR) software was used to quantify band intensities. Three independent experiments were conducted, and a representative blot with relative band intensities for ZapD-GFP, FtsZ, and FtsZ CTV mutants is shown.

Reference:

1. von Hagen J, (2011) Proteomics Sample Preparation. Wiley-Blackwell, NJ, USA.
